# Nocturnal dexmedetomidine alleviates post–intensive care syndrome following cardiac surgery: a prospective randomized controlled clinical trial

**DOI:** 10.1186/s12916-021-02175-2

**Published:** 2021-12-06

**Authors:** Chun-hui Dong, Chao-nan Gao, Xiao-hua An, Na Li, Le Yang, De-cai Li, Qi Tan

**Affiliations:** 1 Department of Cardiac Surgery, Shandong Provincial Hospital Affiliated to Shandong First Medical University, No. 9677 Jingshi Road, Jinan, 250021 Shandong China; 2 Department of Biostatistics, School of Public Health, Cheeloo College of Medicine, Shandong University, Jinan, 250012 Shandong China; 3 Healthcare Big Data Institute of Shandong University, Jinan, 250000 Shandong China; 4 Department of Anesthesiology, Shandong Provincial Hospital Affiliated to Shandong First Medical University, Jinan, 250021 Shandong China; 5 Department of Gynecology, Shandong Provincial Hospital Affiliated to Shandong First Medical University, Jinan, 250021 Shandong China; 6 Department of Cardiology, Shandong Provincial Hospital Affiliated to Shandong First Medical University, Jinan, 250021 Shandong China; 7 Department of Cardiac Surgery, Shandong Provincial Hospital, Cheeloo College of Medicine, Shandong University, Jinan, 250021 Shandong China

**Keywords:** Dexmedetomidine, Post-intensive care syndrome, Cardiac surgery, Critical illness, Prediction

## Abstract

**Background:**

Dexmedetomidine is a sedative agent that may have the potential to reduce the risk of post-intensive care syndrome (PICS). This study aimed to establish whether prophylactic nocturnal dexmedetomidine safely reduces postoperative PICS incidence and to develop an easy-to-use model for predicting the risk of PICS following cardiac surgery.

**Methods:**

This was a single-center, double-blind, randomized, prospective, placebo-controlled trial. Patients undergoing cardiac surgery were randomly assigned (1:1) to dexmedetomidine or placebo (normal saline) groups between January 2019 and July 2020. Dexmedetomidine or a similar volume of saline was administered, with an infusion rate up to 1.2 μg/kg/h until the RASS remained between − 1 and 0. The primary study endpoint was PICS incidence at 6 months follow-up, as defined by cognitive, physical, or psychological impairments.

**Results:**

We assessed 703 individuals for eligibility, of whom 508 were enrolled. Of these, there were 251 in the dexmedetomidine group and 257 in the placebo group that received the trial agent, forming a modified intention-to-treat population. PICS incidence at 6-month follow-up was significantly decreased in the dexmedetomidine group (54/251, 21.5%) relative to the placebo group (80/257, 31.1%) (odds ratio [OR] 0.793, 95% CI 0.665–0.945; *p* = 0.014). Psychological impairment was significantly reduced in the dexmedetomidine group relative to the placebo group (18.7% vs. 26.8%, OR 0.806, CI 0.672–0.967, *p* = 0.029). However, dexmedetomidine treatment was associated with a higher rate of hypotension. A nomogram revealed that age, education, a medical history of diabetes and smoking, dexmedetomidine treatment, postoperative atrial fibrillation, and sequential organ failure assessment scores at 8 h post-surgery were independent predictors of PICS.

**Conclusions:**

Prophylactic nocturnal dexmedetomidine administration significantly reduced PICS incidence by a marked reduction in psychological impairment within a 6-month follow-up period.

**Trial registration:**

ChiCTR, ChiCTR1800014314. Registered 5 January 2018, http://www.chictr.org.cn/index.aspx

**Supplementary Information:**

The online version contains supplementary material available at 10.1186/s12916-021-02175-2.

## Background

Major technical innovations and changes in clinical guidelines have led to the considerable improvement of cardiac intensive care medicine in recent years. These advances have markedly improved the short-term outcomes; however, the long-term improvement of patient quality of life remains one of the most prominent challenges in the field of critical care medicine [1, 2]. The new or worsening impairment of mental health, physical health, and cognition following critical illness have been termed post-intensive care syndrome (PICS) [3]. PICS-related physical impairments can impact roughly 25–80% of survivors and last more than 5 years after discharge, while cognitive deficits impact 4–62% of survivors who experience problems that last for more than 8 years [4, 5]. Different interventions including ICU diary-keeping, cognitive therapy, rehabilitation, and physical and occupational therapy have facilitated symptomatic improvements. However, they have largely failed to improve overall patient long-term quality of life [6–8]. In 2018, the Society of Critical Care Medicine guidelines were updated to incorporate sleep disruption- and immobility-related items in order to better evaluate and monitor patient ICU experiences [9].

Dexmedetomidine is an α2 adrenoceptor agonist that is often administered to patients in the ICU given that it exhibits both sedative and analgesic properties [10]. Perioperative dexmedetomidine treatment has been shown to suppress inflammation, improve postoperative analgesia, and decrease opioid utilization, all of which are probable causes of psychological or cognitive impairment [11, 12]. In our preliminary report, we found that patients administered dexmedetomidine after surgery were less likely to suffer from mental complaints such as anxiety [13]. This property suggests that as our understanding of dexmedetomidine matures, more potential uses of dexmedetomidine for the treatment of one or more domains of PICS may emerge. However, its functional relevance as a tool for the treatment or prevention of PICS is largely unknown.

As psychological, cognitive, or physical impairment levels are highest during the early postoperative days after surgery and dexmedetomidine has the potential to improve these postoperative outcomes [14], we hypothesized that prophylactic nocturnal dexmedetomidine administration may alleviate postoperative PICS in patients undergoing cardiac surgery. We also sought to identify perioperative factors associated with PICS to establish a model capable of predicting PICS risk in cardiac surgery patients.

## Methods

### Study design and oversight

This was a single-center, randomized, prospective, double-blind, parallel-arm trial that was designed to evaluate the safety and efficacy of nocturnal dexmedetomidine administration to patients undergoing cardiac surgery as a means of preventing PICS. The Ethics Committee of Shandong Provincial Hospital approved this study (No. 2018-201), which was registered online prior to recruitment at chictr.org.cn (ChiCTR1800014314). This study was the second stage of the ChiCTR1800014314 protocol. In the first stage of the protocol, the primary endpoint was to delineate the relationship among dexmedetomidine, anxiety, and new-onset postoperative atrial fibrillation (POAF) [13]. We found that anxiety incidence was significantly reduced in the dexmedetomidine group compared to the controlled group (51.6% vs. 67.2%). By collecting cognitive and physical status of patients during follow-up, we also unexpectedly found that dexmedetomidine improved the cognitive and physical impairments of study subjects. As such, we further regenerated the ChiCTR1800014314 protocol, with the new primary outcome having been changed to focus on PICS. Based on the previous results of the first-stage and PICS incidence in other studies [15], we recalculated the necessary sample size for this study. The modified protocol has been registered and filed with the ethics committee of our hospital. Therefore, the current study was an improved version of ChiCTR1800014314. All patients or their legal representatives provided written informed consent to participate in this study, and the safety data associated with this trial were regularly reviewed by an independent Data and Safety Monitoring Board.

### Patient recruitment

Consecutive patients scheduled to undergo elective cardiac surgery associated with a planned ICU stay of over 2 days who were receiving continuous or intermittent sedatives to facilitate safety and comfort were recruited for this study. For full details regarding study inclusion and exclusion criteria, see Additional file 1: Table S1.

### Randomization and masking

Patients were assigned to either a dexmedetomidine or a placebo group at a 1:1 ratio following ICU admission. An independent team that was not involved in study recruitment, assessment, or intervention conducted patient randomization based on a computerized random number generator. The study drug was prepared by the pharmacy or an otherwise uninvolved research associate so that investigators and clinicians were fully blinded to allocation. Selection bias was mitigated by blinding all study personnel to group assignments throughout the 6-month study period until data were unlocked. Identical rehabilitation protocols were conducted in both groups aside from the nocturnal administration of dexmedetomidine, as shown in Additional file 1: Table S2.

### Procedures

#### Sedation goals

This trial sought to achieve light sedation at night unless such sedation was contraindicated or considered to be unsafe by the attending clinician. Such light sedation was defined by a Richmond Agitation and Sedation Scale (RASS) of − 1 to 0 regardless of ventilation status such that patients achieved 6–8 h of sleep at night following surgery.

#### Dexmedetomidine or placebo administration

Dexmedetomidine or placebo (normal saline) were administered at night to patients in the appropriate treatment group as the sole or primary sedating agent utilized to achieve light sedation. Dexmedetomidine or a similar volume of saline was administered without a loading dose at 0.5 μg/kg/h i.v. from 10:00 PM until 6:00 AM the following day (halved at 5:00 am and discontinued at 6:00 am.), with the infusion rate being increased every 30 min when RASS scores were > 0 up to a maximum rate of 1.2 μg/kg/h until the target RASS was achieved because the majority of the safety concerns associated with this drug are dose-related. When RASS scores were ≤ − 2, clinicians were allowed to decrease administration of the study medication by a unit of 0.2 μg/kg/h every 30 min if clinically indicated. RASS scores were assessed at least once every 2 h based upon responses to voices, stimulation, self-control, and vital signs. If a subject was expected to require sustained treatment with sedatives or agitation occurred in treated patients (RASS ≥ 1), the administration of other sedatives such as propofol, opioids, and benzodiazepines was permitted when the effects of dexmedetomidine or placebo were insufficient. When patients were transferred out of the ICU, dexmedetomidine or placebo were administered as required, and were recommended for those suffering from insomnia. Study medication usage extended for at least 3 nights. While the concomitant use of antipsychotic agents to promote sleep induction was discouraged, the use of these agents was permitted at the clinician’s discretion to manage any delirium that occurred, as shown in Additional file 1: Fig. S1 and S2.

### Evaluation of PICS problems

On day 2 after admission, day 7 after surgery, and at 3 and 6 months post-discharge, study personnel blinded to patient group assignments evaluated patient cognitive, physical, and mental health with the MMSE [16], Barthel index [17], Zung Self-Rating Anxiety Scale (SAS) [18], and Zung Self-Rating Depression Scale (SDS) [19] tools, respectively. Assessments were conducted in-person during hospitalization, while they were conducted via an online smartphone-based videoconference during follow-up [20]. Anxiety and depression were selected as representative traits for analyses of mental health challenges, given that anxiety and depression are the most common such manifestations [21], and are five times more common than posttraumatic stress disorder (PTSD) in survivors of critical illness [5]. In light of similar studies and clinical practice, we established that a BI score of < 80 was considered indicative of mild dysfunction and physical impairment [22].

PICS-related impairments were defined using standard threshold values for these respective measurement scales, with an MMSE score ≤ 26 indicating cognitive impairment, a Barthel index score of ≤ 80 indicating disability, and an SAS score and/or SDS > 50 indicating psychological impairment.

### Outcome assessments

PICS incidence at 6 months post-discharge served as the composite primary endpoint for this study. Patients exhibiting at least one of these criteria were considered to have met the primary study endpoint.

Secondary study endpoints included (a) mortality during hospitalization and within 6 months after discharge; (b) PICS incidence and its components at 3 months post-discharge; (c) postoperative ICU stay duration; (d) postoperative hospital stay duration; (e) tracheal intubation time; (f) acute kidney injury(AKI) [23] and POAF [13]; (g) delirium during hospitalization—delirium was defined using the Confusion Assessment Method for the Intensive Care Unit (positive or negative) when the RASS scores were − 2 or higher; (h) postoperative sedative drug use after study drug administration; (i) PICS co-occurrence at 6 months post-discharge—PICS symptom co-occurrence was defined as the new-onset occurrence of two or more of the three PICS elements; (j) sleep quality assessed using Pittsburgh Sleep Quality Index (PSQI) during hospitalization and follow-up; and (k) safety endpoints included hypotension (mean arterial pressure < 60 mmHg during infusion) and severe sinus bradycardia (heart rate < 50 bpm). Supplementary administration of extra fluids or vasoconstrictors to reverse hypotension were also recorded.

### Missing data

**Fig. 1:**
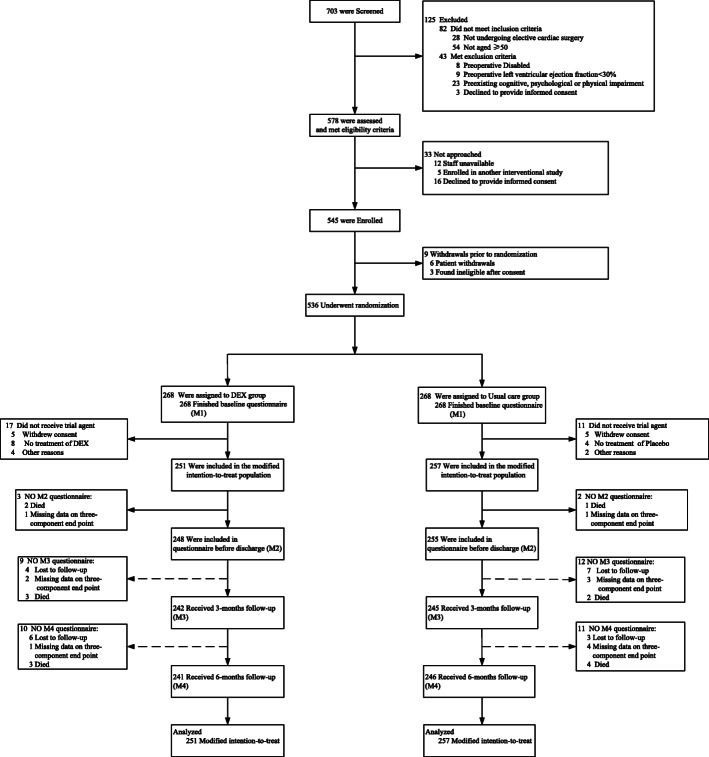
Recruitment, randomization, and analysis populations. Abbreviations are as follows: *DEX* dexmedetomidine

The rate of missing data in our study was low. As some missing data were duplicated, 12 patients in the dexmedetomidine group and 14 in the placebo group ultimately exhibited missing data (Fig. 1). The missing measurement data primarily included SAS, SDS, MMSE, and Barthel index during hospitalization and follow-up, as well as length of ICU stay, length of hospital stay, and tracheal intubation time due in cases of death. For every dataset with missing values, we created imputed datasets and used predictive mean matching multiple imputation at the time of regression modeling to account for missing data at hospitalization or at each time point during follow-up [24]. For all missing data replacement values, we set age and sex as the independent variables in the imputation model. In addition, missing categorical data were replaced by negative results.

### Sample size

Cognitive impairment has previously been shown to affect 30–80% of ICU survivors, while psychological impairment impacts 25–80% of these patients, often for extended periods of time [25, 26]. Specifically, PICS was present in 64% and 56% of survivors at 3 and 12 months, respectively [15]. However, post-ICU sequela rates vary substantially across patient populations, and no comparable studies of the impact of dexmedetomidine on PICS were available. In our previous pilot study, anxiety was significantly reduced by dexmedetomidine administration (51.6% in the dexmedetomidine group and 67.2% in the control group) [13]. We also utilized the MMSE to assess cognitive impairment and the Barthel index to assess physical impairment at the 6-month follow-up time point. PICS incidence was 50.8% (31/61) in the control group and 35.5% (22/61) in the dexmedetomidine group. Based on the literature and our pilot study, we posited that PICS would likely occur in 50% of patients in the placebo group and 35% of patients administered dexmedetomidine at 6 months post-discharge. To achieve 90% power to detect such a difference at a two-tailed significance level of 0.05, we calculated that 227 patients per group would be required. To account for a 15% attrition rate, we targeted enrollment of 261 patients per group, and we ultimately enrolled 268 patients per group.

### Statistical analysis

Primary analyses were conducted on a modified intention-to-treat (MITT) population, shown in Fig. 1. Data are given as means with standard deviations (SDs), median with interquartile ranges (IQRs), or frequencies and proportions, as appropriate. Differences between groups are presented with 95% confidence intervals (CIs). Shapiro-Wilk tests were used to evaluate data normality. Logistic regression analyses were used to assess the impact of treatment on study outcomes using odds ratios (ORs) with 95% CIs. Numeric data were compared via Student’s *t*-tests or Mann-Whitney *U* tests as appropriate. Categorical variables were analyzed with the *χ*^2^ test. MMSE, SAS, Barthel index, and PSQI were analyzed via repeated-measures analyses of variance (RM-ANOVAs) with time as the within factor (*P*_w_), treatment group as the grouping factor (*P*_g_), and interactions identified in the RM-ANOVA (*P*_i_).

Exploratory subgroup analyses of the primary endpoint data were conducted based upon six variables: age (< 65 or ≥ 65), whether or not cardiopulmonary bypass was used, education level (< 9 or ≥ 9 years), PSQI after admission (< 6 or ≥ 6), SOFA score (greater than or below the median [7.14]), and BMI (< 24 or ≥ 24).

Post hoc analyses of PICS risk were divided into two parts. In the first part, 10-fold cross-validation was conducted on the global data of 508 patients (the training cohort of 458 subjects and the validation cohort of 50 subjects) for PICS prediction (Additional file 1: Fig. S3). Univariate logistic regression analyses were used to identify risk factors linked to PICS risk and its components in the training cohort. All baseline variables, such as age, BMI, sex, education, SOFA Score, APACHE score, postoperative atrial fibrillation, dexmedetomidine group status, and previous medical history of hypertension, diabetes and smoking were initially examined as risk factors. If those variables yielded *P* < 0.05, they were incorporated into subsequent multivariate-adjusted binary logistic regression analyses in an effort to identify independent predictors. These independent predictors were then used to prepare a predictive nomogram using the rms package. The area under the ROC curve (AUROC) was used to present the discriminative ability of this nomogram. Calibration curves were generated by plotting predicted vs. actual PICS rates to establish the accuracy of this nomogram. Individual predictors were marked with horizontal lines in the final nomogram. In the second part, we employed multivariate logistic regression analyses to determine dose-response relationships between risk factors and PICS at 6-month follow-up. Dexmedetomidine group status, age, education, diabetes, renal failure, smoking, postoperative atrial fibrillation, and SOFA Score were included as independent variables in the model. All statistical tests were two-sided, and *P* < 0.05 served as the significance threshold. Multiple comparisons testing was not performed, and so all secondary outcome data should be considered exploratory. R for Windows (v.3.42, http://www.r-project.org/) was used for all analyses.

## Results

### Patient population

Between January 2019 and July 2020, 703 consecutive patients were screened for eligibility, of whom 536 met the inclusion/exclusion criteria and were randomly assigned to receive either dexmedetomidine or placebo. Of these, 17 patients in the dexmedetomidine group and 11 patients in the placebo group were excluded during the period of study-drug infusion, thus leaving 251 patients in the dexmedetomidine group and 257 in the placebo group that were enrolled into the study.

At baseline, there were no major differences between the treatment groups with respect to age at enrollment, sex, BMI, education level, previous medical history, or SOFA score after surgery (Table 1). Multi-organ function after surgery was an important consideration because of its potential to aggravate or worsen psychological or physical recovery. Therefore, SOFA and APACHE II scores at 8 h after surgery were collected. SOFA score at 8 h post-surgery was also similar between the treatment groups (7.16 ± 3.1 vs. 7.12 ± 3.01, *p* = 0.899).

**Table 1:** Baseline demographics and characteristics

Variables	Dexmedetomidine group (*n* = 251)	Placebo group (*n* = 257)	*p*
Age, mean ± SD, years	64.84 ± 6.14	64.57 ± 6.22	0.624
BMI, mean ± SD, kg/m^2^	24.98 ± 3.00	24.87 ± 3.26	0.695
Surgery classification, *n* (%)			0.347
without CPB	172(68.5%)	166(64.6%)	
With CPB	79(31.5%)	84(35.4%)	
Men, *n* (%)	166(66.1%)	156(60.7%)	0.204
Education, mean ± SD, years	7.87±2.847	7.46±3.276	0.133
Hypertension, *n* (%)	132(52.6%)	134(52.1%)	0.919
Diabetes, *n* (%)	76(30.3%)	77(30.0%)	0.938
Renal failure, *n* (%)	6(2.4%)	5(1.9%)	0.731
Infarction, *n* (%)	40(15.9%)	38(14.8%)	0.719
Smoking, *n* (%)	75(29.9%)	89(34.6%)	0.252
Alcohol, *n* (%)	61(24.3%)	60(23.3%)	0.8
LVEF, *n* (%)			0.692
≤ 40%	12(4.8%)	13(5.1%)	
41–60%	200(79.7%)	197(76.7%)	
≥ 61%	39(15.5%)	47(18.3%)	
Atrial fibrillation before surgery	13(5.2%)	11(4.3%)	0.633
SOFA score at 8 h after surgery^a^	7.16 ± 3.1	7.12 ± 3.01	0.899
APACHE II at 8 h after surgery^b^	7.706 ± 2.15	7.63 ± 2.012	0.686
PSQI score at admission ^c^	7.1 ± 2.121	7.06 ± 2.161	0.844

*BMI* Body mass index, *CPB* Cardiopulmonary bypass, *COPD* Chronic obstructive pulmonary disease, *LVEF* Left ventricular ejection fraction, *SOFA* Sequential Organ Failure Assessment, *APACHE II* Acute Physiology and Chronic Health Evaluation II, *PSQI* Pittsburgh Sleep Quality Index

^a^SOFA scores are based on six scores, with respiratory, cardiovascular, hepatic, coagulation, renal and neurological system function being scored from 0-4 where higher scores correspond to more severe organ dysfunction

^b^APACHE II scores (0–71) are based upon the values of 12 routine physiological measurements, age, and prior health status, providing a general measurement of disease severity

^c^The PSQI questionnaire consists of 19 scored items assessing seven factors: subjective sleep quality, sleep latency, sleep duration, habitual sleep efficiency, sleep disturbances, use of sleeping pills, and daytime dysfunction. All factors are scored from 0–3, with higher scores corresponding to poorer quality of sleep

### Primary and secondary study endpoints

PICS at 6-month follow-up was the primary study outcome and was observed in 21.5% (54/251) of patients in the dexmedetomidine group and 31.1% (80/257) of patients in the placebo group (OR 0.793; 95% CI, 0.665–0.945; *p* = 0.014), shown in Table 2. Of the assessed patients, 10 (3.98%) in the dexmedetomidine group and 17 (6.61%) in the placebo group exhibited cognitive impairment (OR 0.792, CI 0.585–1.073, *p* = 0.186), while 7 (2.79%) and 13 (5.06%), respectively, exhibited disability (OR 0.769, CI 0.551–1.074, *p* = 0.188), and 47 (18.7%) and 69 (26.8%), respectively, exhibited psychological impairment (OR 0.806, CI 0.672–0.967, *p* = 0.029).

**Table 2:** Primary and secondary endpoints

Variables	Dexmedetomidine group (*n* = 251)	Placebo group (*n* = 257)	OR (95%CI)	*P* value
**Primary endpoint**				
PICS at 6-month after discharge ^#^	54(21.5%)	80(31.1%)	0.793(0.665–0.945)	0.014
Individual component of the primary end point				
Cognitive impairment by MMSE ^a^	10(4.0%)	17(6.6%)	0.792(0.585–1.073)	0.186
Psychological impairment by SAS and SDS ^b,c#^	47(18.7%)	69(26.8%)	0.806(0.672–0.967)	0.029
Physical impairment by Barthel index ^d^	7(2.8%)	13(5.1%)	0.769(0.551–1.074)	0.188
**Secondary endpoints**				
Mortality in hospitalization, No (%)	2(0.8%)	1(0.4%)	1.521(0.306–7.522)	0.549
Mortality within 6 months, No (%)	3(1.2%)	4(1.6%)	0.884(0.463–1.688)	0.727
PICS at 3 months after discharge ^#^	79 (31.5%)	110 (42.8%)	0.792(0.668–0.938)	0.008
Cognitive impairment*	18(7.2%)	29(11.3%)	0.802(0.628–1.022)	0.11
Psychological impairment^*,#^	58(23.1%)	84(32.7%)	0.799(0.671–0.951)	0.016
Disability^*,#^	16(6.4%)	34(13.2%)	0.716(0.579–0.885)	0.01
ICU stay	3.0(2.0,4.0)	3.0(2.0,4.0)	-	0.642
Length of hospital stay	8(7.0,10.0)	8(7.0,11.0)	-	0.157
Tracheal intubation time	13(9,16)	13(9,17)	-	0.346
Retracheal intubation	2(0.8%)	5(1.9%)	0.704(0.437–1.134)	0.267
Acute kidney injury	3(1.2%)	2(0.8%)	1.267(0.432–3.721)	0.634
Delirium	19 (7.6%)	31 (12.1%)	0.796(0.629–1.008)	0.089
Postoperative atrial fibrillation	57 (22.7%)	80 (31.1%)	0.817(0.684–0.975)	0.033
Safety outcomes				
Any adverse event occurred ^#^	45(17.9%)	26(10.1%)	1.443(1.050–1.985)	0.011
Hypotension after treatment initiation ^#^	32(12.7%)	17(6.6%)	1.507(1.016–2.235)	0.019
Bradycardia after treatment	17(6.8%)	12(4.7%)	1.236(0.795–1.923)	0.307
Extra fluid intervention	13(5.2%)	6(2.3%)	1.625(0.834–3.168)	0.091
Extra vasoconstrictor intervention	7(2.8%)	4(1.6%)	1.4(0.638–3.074)	0.34

Data are given as means ± standard deviation (SD) or mean (IQR) for measurement variables and number of patients (*n*) and percentages (%) for categorical variables. *OR* odds ratio, *CI* confidence interval, *PICS* Post-intensive care syndrome, *MMSE* Mini-Mental State Examination, *SAS* Zung’s Self-Rating Anxiety Scale, *SDS* Self-Rating Depression Scale

^a^The Mini-Mental State Examination (MMSE) uses a 30-point scale to evaluate cognitive function based upon tests of patient orientation, concentration, attention, verbal memory, naming, and visuospatial skills. Scores of < 27 points are consistent with potential cognitive impairment

^b^Zung’s SAS is a 20-item questionnaire with scores ranging from 20–80 points. A score of > 50 points is consistent with potential generalized anxiety disorder

^c^Zung’s SDS contains 20 items using a 4-point Likert scale. The raw sum score of the SDS ranges from 20 to 80 but results are usually presented as the SDS Index, which is obtained by expressing the raw score is converted to 100 points scale. The SDS cut-off points for depression were > 50 based on literature

^d^The Barthel Index is a scale that assesses the ability to perform particular activities of daily living. This index consists of 10 tasks that are scored from 0 to 100, with higher scores corresponding to greater mobility. A score of < 80 is consistent with potential physical impairment

*Rates of cognitive impairment, anxiety, and disability was at 3 months’ follow-up

#Variables with significant difference, *p* < 0.05

With respect to secondary outcomes, mortality within 6 months of discharge occurred in 1.2% (3/251) of patients in the dexmedetomidine group and in 1.6% (4/257) of patients in the placebo group, with no significant differences. Rates of PICS at 3 months post-discharge in the dexmedetomidine group was 31.5% (79/251), versus 42.8% (110/257) in the placebo group (OR, 0.792; 95% CI, 0.668–0.938; *p* = 0.008). Postoperative atrial fibrillation was observed in 57 (22.7%) patients in the dexmedetomidine group and 80 (31.1%) in the placebo group, with a significant difference between groups. There were no differences in the median mechanical ventilation duration, duration of ICU stay, hospital stay, or delirium when comparing groups.

**Fig. 2:**
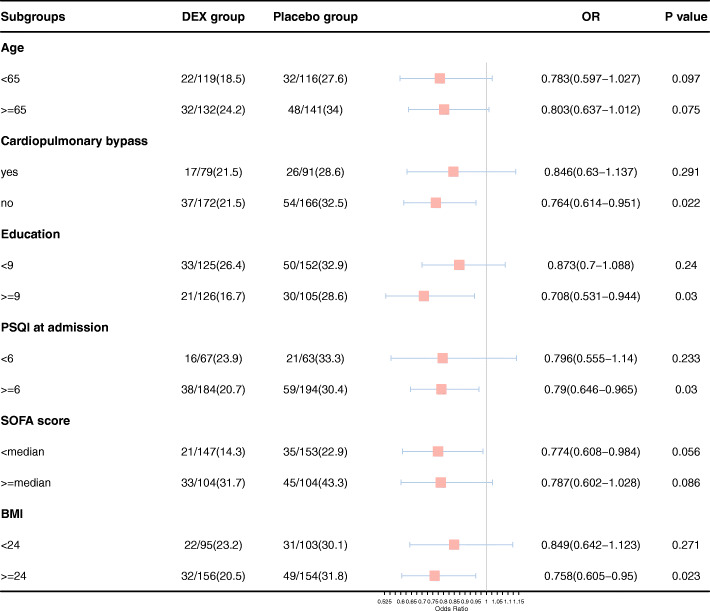
Subgroup analyses of PICS incidence rates. Abbreviations are as follows: *PSQI* Pittsburgh Sleep Quality Index, *SOFA* sequential organ failure assessment, *BMI* body mass index

The results of exploratory subgroup analyses are shown in Fig. 2. PICS incidence rates differed between the two treatment groups in subsets of patients that underwent surgery without CPB (*p* = 0.022), had an education level of ≥ 9 years (*p* = 0.03), exhibited PSQI scores ≥ 6 (*p*=0.03), and had a BMI ≥ 24 (*p* = 0.023).

**Fig. 3:**
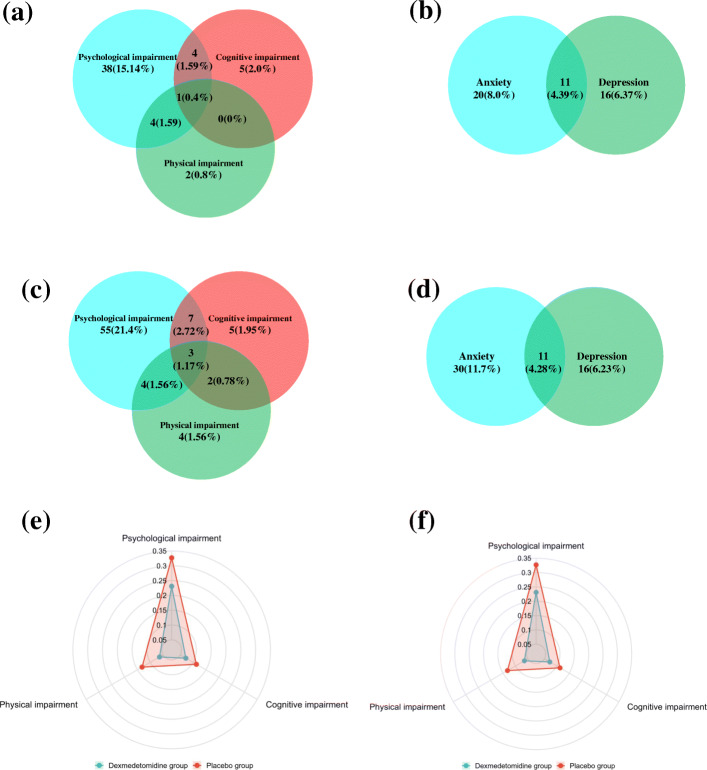
Co-occurring PICS symptoms at 3- and 6-month follow-up. The proportion of patients with PICS-related symptoms in each domain at 6-month and 3-month follow-up are shown in **a** and **c**, respectively. Cognitive impairment is represented by a red circle, while disability in activities of daily living is represented by a green circle, and psychological impairment is represented by a blue circle. The overlap between these circles represents the co-occurrence of two or three of these problems. The proportion of psychological impairment associated with anxiety and depression at the 6-month and 3-month follow-up time points is shown in **b** and **d**, respectively. Blue and green circles correspond to anxiety and depression, respectively. Radar graphs were used to illustrate the incidence of each PICS domain at 6-month follow-up (**e**) and 3-month follow-up (**f**). Dexmedetomidine treatment is represented by a blue line while placebo treatment is represented by a red line

At the 6-month follow-up time point, a single PICS-related impairment was evident in 45 (17.9%) patients in the dexmedetomidine group and 64 (24.9%) in the placebo group, whereas 9 (3.6%) and 16 (6.23%) of patients in these respective groups exhibited two or more PICS-related impairments, as shown in Fig. 3.

With respect to safety outcomes, the incidence of adverse events (hypotension or bradycardia) differed significantly between these groups, with 45 instances (17.9 %) in the dexmedetomidine group and 26 (10.1%) in the placebo group (OR, 1.443; 95% CI, 1.05–1.985; *p* = 0.011). Specifically, hypotension rates were significantly increased in the dexmedetomidine group, affecting 32 (12.7%) of these patients versus just 17 (6.6%) in the placebo group (OR, 1.507; 95% CI, 1.016–2.235; *p* = 0.019), while bradycardia did not differ between groups, with 17 (6.8%) patients in dexmedetomidine group and 12 (4.7%) patients in the placebo group.

### Study drug infusion

All patients in the dexmedetomidine group were infused with this study drug for an average of 5 [4-5] nights. Of these patients, 12 (4.8%) patients were treated with propofol and 34 (13.5%) were treated with alprazolam to improve sedation. In the placebo group, 39 (15.2%) patients were administered propofol and 96 (37.4%) were administered alprazolam. Olanzapine was the preferred agent for postoperative relief of delirium and was administered to 19 (7.6%) in the dexmedetomidine group and 37 (14.4%) in the placebo group (*p* = 0.014), as shown in Additional file 1: Table S3.

### Predictive nomogram construction and dose-response curves

**Fig. 4:**
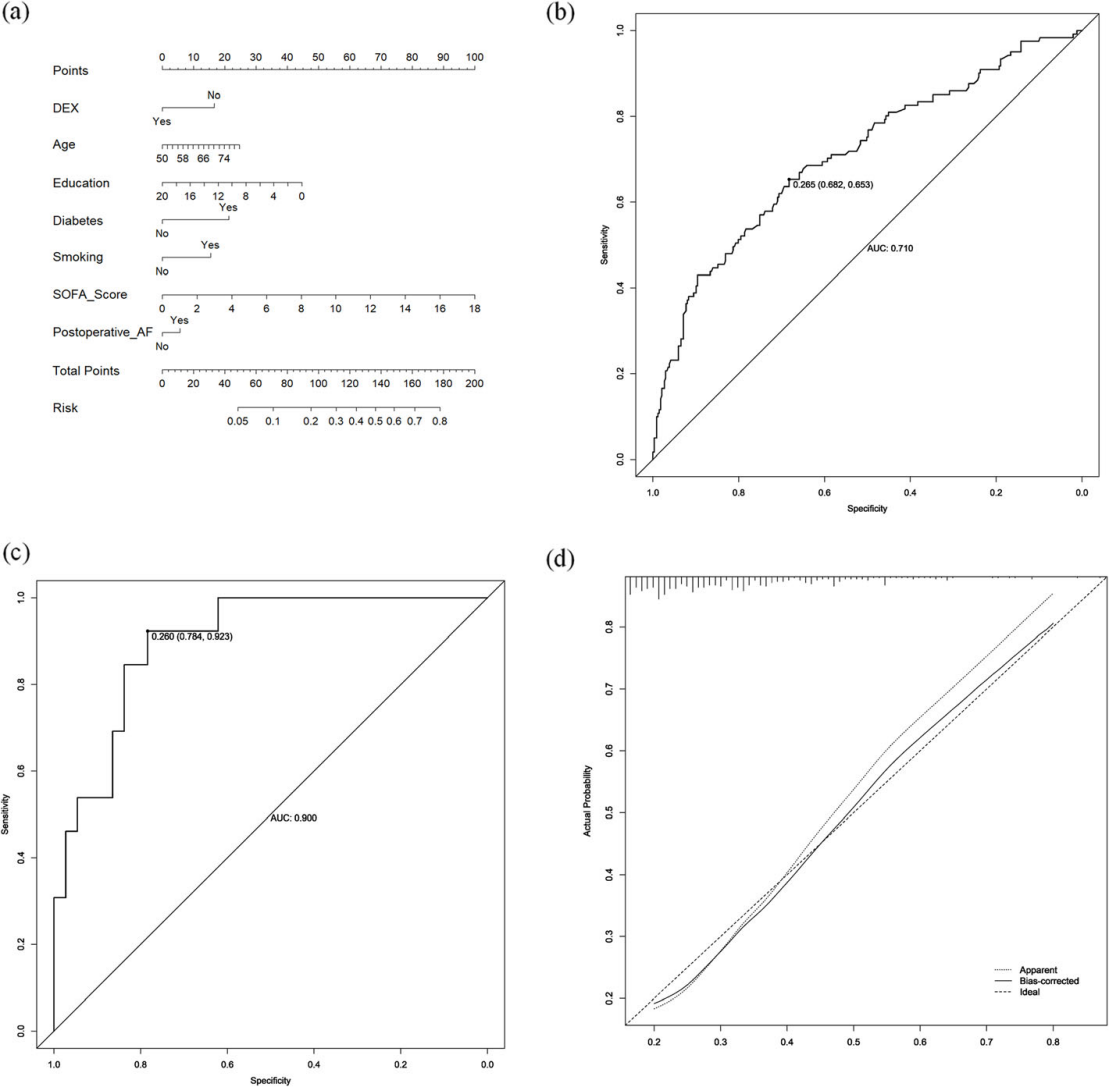
Nomogram model for the prediction of individual PICS risk in patients at the 6-month follow-up time point. **a** Nomogram for PICS; a comparison of area under the receiver operating characteristic curve (AUROC) values for the prediction of PICS in the training cohort (**b**) and validation cohort (**c**). **d** Calibration curves for nomogram-based assessments of the training cohort. Abbreviations are as follows: *DEX* dexmedetomidine, *AF* atrial fibrillation

**Fig. 5:**
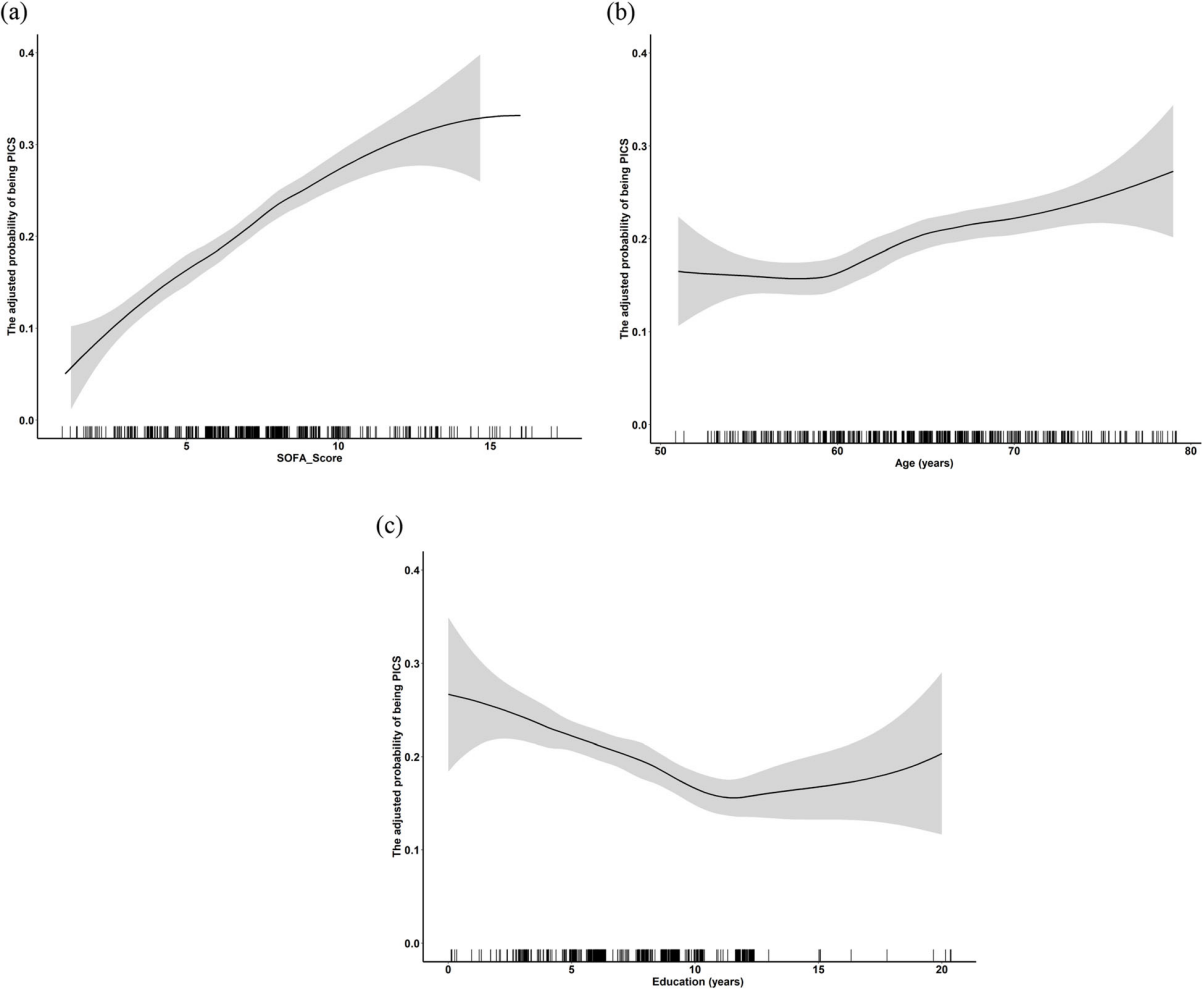
Association between numerical predictive variables (SOFA score, age, and education) and PICS incidence at 6-months follow-up. **a** Association between SOFA score and PICS incidence at 6 months’ follow-up. **b** Association between age and PICS incidence at 6 months’ follow-up. **c** Association between education and PICS incidence at 6 months’ follow-up. The black lines correspond to the association, while the shaded area corresponds to the 95% CI. Abbreviations are as follows: *SOFA* Sequential Organ Failure Assessment

Univariate and multivariate analyses led to the identification of seven variables that were independent predictors of PICS incidence at 6 months post-discharge in cardiac surgery patients, including advanced age, fewer years of education, a medical history of diabetes and smoking, higher SOFA scores at 8 h post-surgery, postoperative atrial fibrillation, and a lack of dexmedetomidine administration (Additional file 1: Table S4). Based on these results, a predictive nomogram incorporating these variables was constructed to gauge globality and each domain of PICS. ROC analyses revealed the AUROC for this nomogram in the training and validation cohorts to be 0.71 and 0.9, as shown in Fig. 4. Calibration plots further exhibited good agreement between actual and predicted odds of PICS. The dose-response curves between SOFA score and PICS incidence exhibited a significant positive correlation, with a similar tendency for age and PICS incidence, as shown in Fig. 5.

## Discussion

This study is the first randomized study to demonstrate the benefits of dexmedetomidine infusion as they pertain to PICS incidence in patients undergoing cardiac surgery. This held true even after dexmedetomidine administration was terminated. However, patients treated with dexmedetomidine exhibited higher rates of adverse events relative to the placebo group, with hypotension being the most common such event. In addition, we developed a valuable predictive nomogram capable of predicting individual patient PICS risk that can be readily utilized in clinical practice.

The reported epidemiology of PICS among critical illness survivors differs greatly in different studies. One study following 196 survivors of critical illnesses for a median of approximately 3 years found that 52% experienced prolonged psychiatric symptoms, while another study of acute respiratory distress syndrome survivors found that of 102 patients who underwent neuropsychiatric evaluation 1 year following hospitalization, 36% exhibited depression and 62% exhibited anxiety [27, 28]. In the present study, however, we observed relatively low rates of PICS in critical illness patients following cardiac surgery. There may be several reasons for these differences. For one, these prior studies often enrolled critically ill patients undergoing medical therapy who were suffering from shock or respiratory failure, whereas we focused on patients undergoing cardiac surgery who were free of preexisting cognitive or physical impairments. Surgical resolution of underlying disease and related symptoms may also aid in the recovery of associated psychological or physical symptoms. Second, as this was a randomized controlled trial, we have excluded certain PICS-related susceptibility factors such as parkinsonism, schizophrenia, unplanned ICU admission, and hepatic dysfunction. As such, these differences in study populations may account for the differences in PICS incidence. Most studies that have previously been performed for PICS did not include patients undergoing cardiac surgery. Therefore, our study is the first to report the prevalence of PICS in cardiac surgery patients.

With respect to safety, studies of patients undergoing cardiac surgery have shown that dexmedetomidine is not linked to significant hypotension nor does it differ from other sedatives with respect to vasopressor requirements [29]. Yoanna et al. reported that the nocturnal administration of low-dose dexmedetomidine (0.2–0.7 mcg/kg/min) reduced delirium without increasing hypotension in critically ill adults [30]. Su et al. also determined that dexmedetomidine infusion (0.1mcg/kg/min) bolstered psychological recovery including delirium without hypotension [14]. In our study, a larger infusion rate of dexmedetomidine was utilized, and other supplemental sedatives were permitted and should be beneficial as a means of reducing PICS incidence. However, we observed higher rates of dexmedetomidine-induced hypotension during the early postoperative period. This may be due to the larger infusion rate of dexmedetomidine administered to ensure target sedation and to unstable hemodynamics in cardiac surgery patients. While we did observe differences in adverse event frequencies between the two groups in the present study, there were no significant differences between these groups with respect to extra interventions beyond withdrawal.

Prior studies have provided evidence regarding the potential benefits of dexmedetomidine as a mediator of physical and psychological recovery; it remained unclear whether these long-term benefits were attributable to the prophylactic administration of dexmedetomidine [31]. Herein, we found that dexmedetomidine administration significantly reduced PICS incidence not only at 3 months post-surgery, but also decreased such incidence up to 6 months post-surgery. The long-term effects of this treatment may be attributable to both better short-term rapid effects and indirect effects. First, accelerated short-term recovery helped to achieve better long-term outcomes in these patients. We believe that any prophylactic pharmacological interventions should be initiated at an early time point, as the prevalence of cognitive disorders including anxiety and delirium is highest early after surgery. In addition, sleep problems on the first night after surgery are reported by > 40% of patients and often continue for multiple days following surgery [32]. Nocturnal light sedation is encouraged, as it can improve postoperative sleep quality, restore normal sleep-wake cycles, and thereby decrease rates of postoperative psychological impairment, given that dexmedetomidine facilitates the promotion of sleep via endogenous pathways [33]. Secondly, dexmedetomidine may reduce postoperative PICS incidence owing to its indirect multi-organ effects. Some studies have demonstrated that dexmedetomidine significantly decreased hypoxemia in elderly patients after surgery and prevented delirium not just during drug infusion but for up to 3 days postoperatively, while studies have also shown that dexmedetomidine can provide analgesia by acting on the α2 adrenergic receptors in the spinal cord [14, 34]. Other studies suggested that dexmedetomidine was able to exert renoprotective activity through its anti-inflammatory effects [35], and dexmedetomidine administration helped to reduce excessive supplemental sedative or analgesic use, each of which has the potential to increase the risk of postoperative psychological and cognitive impairment [36]. In our post hoc subgroup analyses, dexmedetomidine was found to be useful in more severely ill patients, individuals suffering from preoperative sleep disorders, and those with a higher BMI. In light of these results, we believe that further study of the untapped potential of dexmedetomidine in different patient populations and surgical procedures is warranted.

In addition to a lack of dexmedetomidine administration, we also identified other variables that were associated with a higher risk of postoperative PICS. The association between aging and the impairment of physical, cognitive, and psychological functionality is not surprising, and cardiovascular surgery may further exacerbate such impairment. Marra et al. previously demonstrated that there was a 1.6-fold higher rate of PICS among individuals with lower levels of education relative to more educated patient populations. Many studies have confirmed that nicotine addicts often suffer from psychological problems such as depression and anxiety [37, 38], and diabetes is also related to mild cognitive decrements [39]. Surprisingly, we found that SOFA scores were very accurate predictors of PICS incidence. Most current intensive care-based scoring systems exclude patients undergoing cardiac surgery due to a lack of available postoperative risk stratification tools for these patients. Abraham et al. found that SOFA scores were able to more effectively predict hospital- and ICU-related mortality as compared to APACHE scores [40]. Martin et al. also explored the relevance of SOFA scores at the time of ICU admission and found them to be related to 28-day mortality following cardiac arrest [41]. This study was the first to validate a correlation between SOFA scores and PICS incidence. We found that the higher SOFA scores were positively associated with increased PICS incidence up to the 6-month follow-up time point.

There are multiple limitations to this analysis. First, as a syndrome, it is hard to define PICS in a standardized manner, and the criteria for selecting tools to analyze PICS are ambiguous. While we utilized widely accepted scales to evaluate cognitive impairment and disability, the heterogeneity of these scales still limits the synthesis and interpretation of study findings within this field and may contribute to potential bias associated with selective reporting. Second, we only included patients undergoing cardiac surgery in our study cohort, making it less appropriate for clinical practice when analyzing patients with other diseases and thus limiting the generalizability of our study. Third, patients were permitted to undergo treatment with additional sedatives if necessary. Therefore, some of the alleviation of PICS may have been associated with these supplementary sedatives. Fourth, the sample size in our study is large for a randomized controlled trial; however, it is likely insufficient for prediction analysis. Given that our randomized controlled trials can provide a rigorous design and good balance of baseline data, and AUROC values were presented of 0.710 in the training cohort, and 0.9 in the validation cohort, the 508 patients enrolled in our study is a fairly large sample size study in the domain of PICS. We believed that as the sample size increases, the predictive nomogram could be more meaningful with respect to its clinical significance.

## Conclusions

We found that the prophylactic nocturnal administration of dexmedetomidine can significantly lower rates of PICS in patients undergoing cardiac surgeries at 6 months post-discharge as evidenced by a marked reduction in psychological impairment. We also found that a combined evaluation of patient dexmedetomidine treatment status and risk factors including education, postoperative SOFA scores, and preoperative cognitive, psychological, and physical impairment enabled us to better predict a given individual’s risk of developing PICS. Whether this novel dexmedetomidine treatment approach is associated with better long-term outcomes for different patient populations or surgical procedures, however, requires further study.

## Supplementary information

**Additional file 1. **Supplemental description of methods and results, including Tables S1-S4 and Figure S1-S4. **Table S1.** [Study eligibility criteria]. **Table S2**. [Rehabilitation treatment procedures]. **Table S3.** [Post-Randomization Sedative, Analgesic and Adjunct Medications]. **Table S4.** [Univariate and multivariable analyses of the associations between baseline variables and PICS at 6-month after discharge in the training cohort]. **FigS1.** [Nocturnal dexmedetomidine treatment algorithm]. **FigS2.** [Nocturnal Placebo treatment algorithm]. **FigS3.** [ Analysis flowchart for the prediction of PICS at 6 months post-surgery in the training cohort]. **FigS4.** [Nomogram model and AUROC for the prediction of the individual risk of psychological impairment in patients at 6-month follow-up].

**Additional file 2.**

**Additional file 3.**

**Additional file 4.**

**Additional file 5.**

**Additional file 6.**

**Additional file 7.**

**Additional file 8.**

## Data Availability

As required.

## Abbreviations

CI: Confidence interval
DEX: Dexmedetomidine
ICU: Intensive care unit
IQRs: Interquartile ranges
MITT: Modified intention-to-treat
MMSE: Mini-mental state examination
OR: Odds ratio
PICS: Post-intensive care syndrome
PSQI: Pittsburgh Sleep Quality Index
PTSD: Posttraumatic stress disorder
RASS: Richmond Agitation and Sedation Scale
SAS: Zung Self-Rating Anxiety Scale
SOFA Score: The Sequential Organ Failure Assessment score
