# Supplementary material for: Nocturnal dexmedetomidine alleviates post–intensive care syndrome following cardiac surgery: a prospective randomized controlled clinical trial
Source: BMC Med. 2021 Dec 6;19:306. doi: 10.1186/s12916-021-02175-2 (PMC8647374; doi:10.1186/s12916-021-02175-2)
Supplement: Supplementary file 1 — Additional file 1. Supplemental description of methods and results, including Tables S1-S4 and Figure S1-S4. Table S1. [Study eligibility criteria]. Table S2. [Rehabilitation treatment procedures]. Table S3. [Post-Randomization Sedative, Analgesic and Adjunct Medications]. Table S4. [Univariate and multivariable analyses of the associations between baseline variables and PICS at 6-month after discharge in the training cohort]. FigS1. [Nocturnal dexmedetomidine treatment algorithm]. FigS2. [Nocturnal Placebo treatment algorithm]. FigS3. [ Analysis flowchart for the prediction of PICS at 6 months post-surgery in the training cohort]. FigS4. [Nomogram model and AUROC for the prediction of the individual risk of psychological impairment in patients at 6-month follow-up]. [file 12916_2021_2175_MOESM1_ESM.docx]

**Supplementary Appendix**

This appendix has been provided by the authors to provide readers with additional information about this study.

**Nocturnal Dexmedetomidine alleviates** **Post–intensive care syndrome following cardiac surgery: a prospective randomized controlled clinical trial**

Chunhui Dong^1#^, Chaonan Gao^3,4#^, Xiaohua An^5#^, Na Li^6^, Le Yang^7^, Decai Li^1^, Qi Tan ^1,2*^

**TABLE OF CONTENTS**

Table S1. Study eligibility criteria Page 2

Table S2. Rehabilitation treatment procedures Page 3-4

Table S3. Post-Randomization Sedative, Analgesic and Adjunct Medications Page 4

Table S4. Univariate and multivariable analyses of the associations between baseline variables and PICS at 6-month after discharge in the training cohort Page 5

Figure S1. Nocturnal dexmedetomidine treatment algorithm Page 6-7

Figure S2. Nocturnal Placebo treatment algorithm Page 8-9

Figure S3. Analysis flowchart for the prediction of PICS at 6 months post-surgery in the training cohort Page 10

Figure S4. Nomogram model and AUROC for prediction of the individual risk of psychological impairment in patients at 6-month follow-up Page 11-13

**Table S1: Study eligibility criteria**

**Full inclusion criteria**

1. Patient age > 50 years;

2. Patients underwent scheduled cardiac surgery under general anesthesia with a planned ICU stay of at least 2 days;

3. The attending physician expected the patient to require sustained treatment with sedatives in order to ensure their comfort, safety, and sleep quality;

4. Patients or their legal representatives provided written informed consent.

**Full exclusion criteria**

1. Patients that underwent emergency cardiac surgery;

2. New York Heart Association class Ⅳor those with an ejection fraction ˂ 30%;

3. Patients with preexisting cognitive impairment as indicated by an MMSE score of ≤ 26, a history of schizophrenia, epilepsy, or parkinsonism;

4. Patients with a proven or suspected history of severe stroke or other pathology that may result in anxiety (SAS>50), depression (SDS>50), or permanent or prolonged weakness (i.e., Barthel score ˂80);

5. Patients with preoperative sick sinus syndrome, severe sinus bradycardia (heart rate < 50 bpm), or atrioventricular block of second degree or above without pacemaker;

6. Patients with any known sensitivities to any of the study medications or the components of propofol (egg, soy, or peanut proteins), or patients with an allergy to α2-adrenergicagonist or opioids;

7. Patients suffering from preoperative coma;

8. Patients suffering from brain injury or undergoing neurosurgery;

9. Patients suffering from serious hepatic dysfunction (Child-pugh class C) or renal dysfunction (undergoing dialysis before surgery);

10. Patients for whom death is believed to be imminent or inevitable during admission and for whom either the attending physician, patient, or substitute decision maker is not committed to active treatment;

11. Patients previously recruited for other clinical trials;

12. Patients affected by blindness, deafness, an inability to comprehend standard Chinese, or active substance abuse and psychotic disorders were also excluded to facilitate reliable follow-up.

**Table S2: Rehabilitation treatment procedures**

Aside from the nocturnal sedation strategy, similar rehabilitation treatment protocols were used to treat patients in both the dexmedetomidine and standard care groups.

Preoperative treatment

• Preoperative assessment, education, and psychological counselling were conducted by trained research personnel

• EPO therapy was provided from the time of hospital admission

• Carbohydrate-containing beverage intake was reduced within 2 h prior to anesthetization

• No preoperative sedative or anticholinergic drug use

• Antibiotic prophylaxis was administered within 1 h of anesthesia

Intraoperative treatment

• Fast-track cardiac anesthesia was achieved using short-acting narcotic and sedative agents

• Optimization of CPB: total priming fluid reduction to < 1500ml, modified ultrafiltration, and albumin infusion were used to maintain stable plasma colloid osmotic pressure

• Lung protection strategy: low tidal volume (6-7 ml/kg) ventilation, positive end-expiratory pressure (5 mmHg), lung recruitment maneuver

• Goal-directed fluid management was conducted to optimize stroke volume guided by TOE

• Blood conservation measures: cell saver, antiplasmin agent, and TEG monitor

Postoperative treatment

• PONV prevention (ondansetron)

• EPO therapy

• Early oral intake after tracheal extubation

• Early removal of drainage tube

ABCDEFGH treatment. ABCDE is composed of: A, airway management, assess, prevent, and manage pain, with routine postoperative analgesia (primarily sufentanil) being administered intravenously via a patient-controlled analgesic pump to facilitate continuous intravenous infusion; B, breathing trials, including daily interruptions of mechanical ventilation, spontaneous awakening trials, and spontaneous breathing trials; C, choice of analgesia and sedation, coordination of care, and communication; D, delirium assessment, prevention, and management; and E, early mobility and exercise. Furthermore, FGH correspond to: F, family involvement, follow-up referrals, and functional reconciliation; G, good handoff communication; and H, handout materials regarding PICS.

**Table S3 Post Randomization** **Sedative, Analgesic, and Adjunct Medications**

| Medication | Dexmedetomidine group(n=251) | Placebo group(n=257) | P |
| --- | --- | --- | --- |
| Dexmedetomidine |  |  |  |
| Patients n (%) | 251(100) | 257(100) | -* |
| Median duration of infusion [IQR] d | 5[4-5] | 4[3-5] | -* |
| Propofol |  |  |  |
| Patients n (%) | 12(4.8) | 39(15.2) | ˂0.001 |
| Median duration of infusion [IQR] d | 0[0-0] | 0[0-2] | ˂0.001 |
| Alprazolam |  |  |  |
| Patients n (%) | 34(13.5) | 96(37.4) | ˂0.001 |
| Median duration of infusion [IQR] d | 0[0-0] | 0[0-3] | ˂0.001 |
| Olanzapine |  |  |  |
| Patients n (%) | 19(7.6) | 37(14.4) | 0.014 |
| Median duration of infusion [IQR] d | 0[0-0] | 0[0-0] | 0.013 |
| Sufentanil |  |  |  |
| Patients n (%) | 232 (92.4) | 229 (89.1) | 0.196 |
| Median diose of infusion | 50[50-50] | 50[50-50] | 0.855 |

Data are numbers (%) or medians (interquartile range)

*Corresponding drugs were different in two groups, therefore, we did not analyze the statistical difference

**Table S4:** **Univariate and multivariable analyses of the associations between baseline variables and PICS at 6-month following-up in the training cohort**

| Variable | Univariable | | Multivariable |  |
| --- | --- | --- | --- | --- |
|  | OR (95%CI) | p | OR (95%CI) | p |
| Age | 1.023  (0.986-1.061) | 0.015 | 1.023  (0.986-1.061) | 0.23 |
| Education | 0.914  (0.85-0.982) | 0.014 | 0.93  (0.864-1.001) | 0.055 |
| Dexmedetomidine intervention | 1.7  (1.112-2.599) | 0.014 | 1.725  (1.096-2.717) | 0.019 |
| Diabetes | 1.84  (1.19-2.843) | 0.006 | 2.056  (1.279-3.305) | 0.003 |
| Smoking | 1.778  (1.149-2.75) | 0.01 | 1.769  (1.109-2.822) | 0.017 |
| Postoperative atrial fibrillation | 1.586  (1.007-2.499) | 0.047 | 1.3  (0.795-2.125) | 0.296 |
| SOFA score after 8 h after surgery | 1.198  (1.116-1.288) | <0.001 | 1.28  (1.177-1.392) | <0.001 |

SAS: Zung’s self-rating anxiety scale; SOFA score: Sequential Organ Failure Assessment score; CI, confidence interval; OR: Odds Ratio. Univariate analyses were conducted via chi-squared tests or Fisher’s exact test. Multivariate logistic regression analyses incorporating age, education, dexmedetomidine intervention, SAS before surgery, and SOFA score at 8 h post-surgery was conducted, as these variables yielded P-values < 0.15 in univariate analyses.

**Figure S1. Nocturnal dexmedetomidine treatment algorithm**


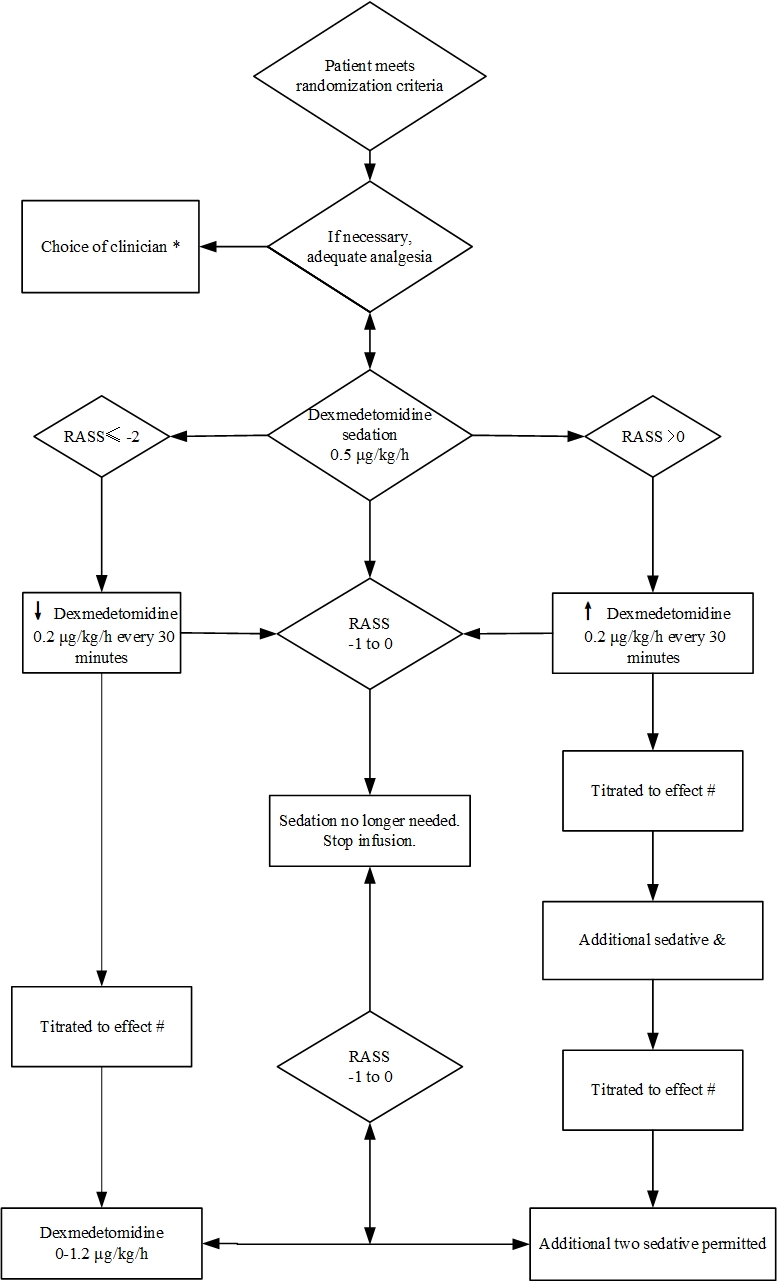


Illustration: Dexmedetomidine was administered at night to patients as the sole or primary sedating agent utilized to achieve light sedation defined by a Richmond Agitation and Sedation Scale (RASS) of −1 to 0. Dexmedetomidine was administered without a loading dose at 0.5 μg/kg/h i.v. from 10:00 PM until 6:00 AM the following day, with the infusion rate being increased every 30 min when RASS scores were ≥ 0 up to a maximum rate of 1.2 μg/kg/h until the target RASS was achieved. When RASS scores were ≤ -2, the study drug infusion rate was decreased. When patients were transferred out of the ICU, dexmedetomidine was administered as required, and was recommended for those suffering from insomnia.

When dexmedetomidine had been titrated to the maximum dose, propofol or midazolam i.v. administration was permitted. While the concomitant use of antipsychotic agents to promote sleep induction was discouraged, the use of these agents was permitted at the clinician’s discretion to manage any delirium that occurred.

*：Analgesic drugs can be provided in the form of infusions or boluses, and can be administered continuously as required throughout the study;

#：Sedative drugs are adjusted every 30 minutes to the lowest effective dose;

&: Benzodiazepines and propofol are permitted.

**Fig S2. Placebo treatment algorithm**


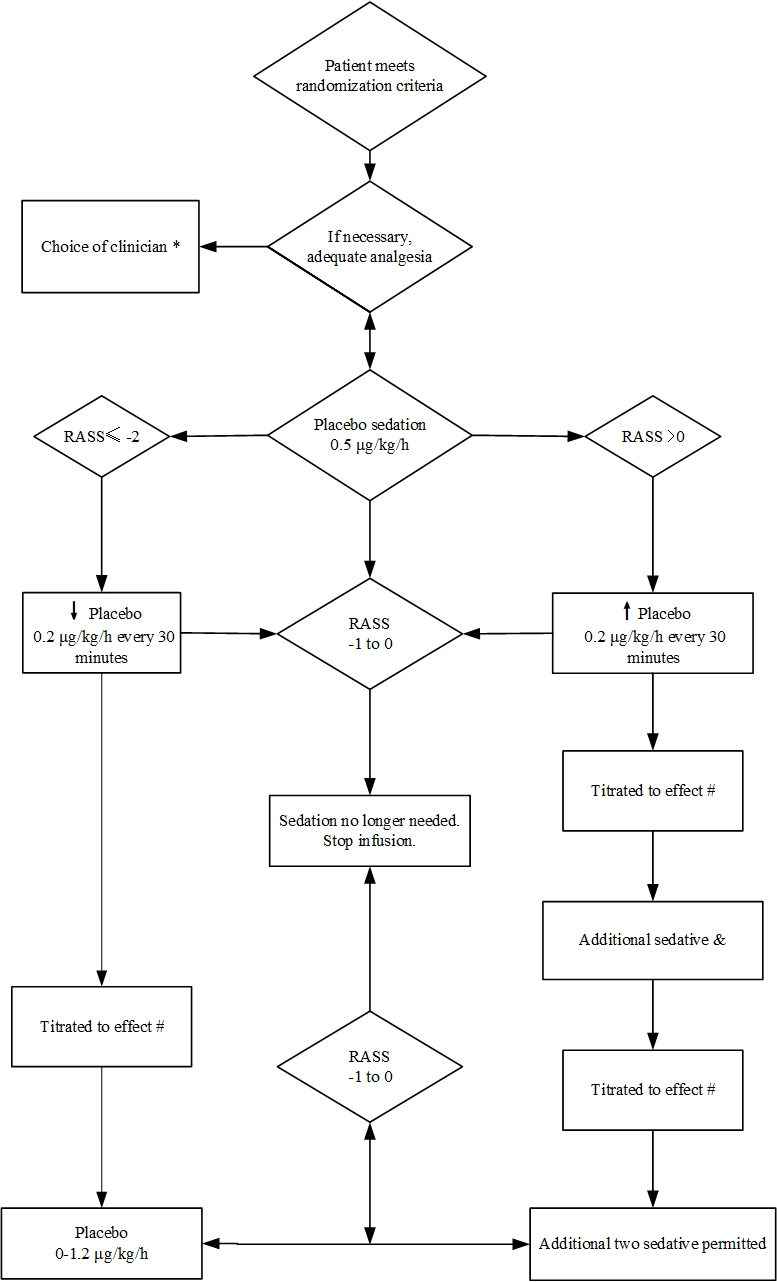


Illustration: Placebo sedation was administrated in order to achieve a target RASS score in the same range as described above. For these patients, additional sedative selection was left to the discretion of the attending clinician.

*：Analgesic drugs can be provided in the form of infusions or boluses, and can be administered continuously as required throughout the study;

& Benzodiazepines and propofol are permitted.

#：Sedative drugs are adjusted every 30 minutes to maintain RASS score between -1 and 0.

**Figure S3. Analysis flowchart for the prediction of PICS at 6-month post-surgery in the training cohort**


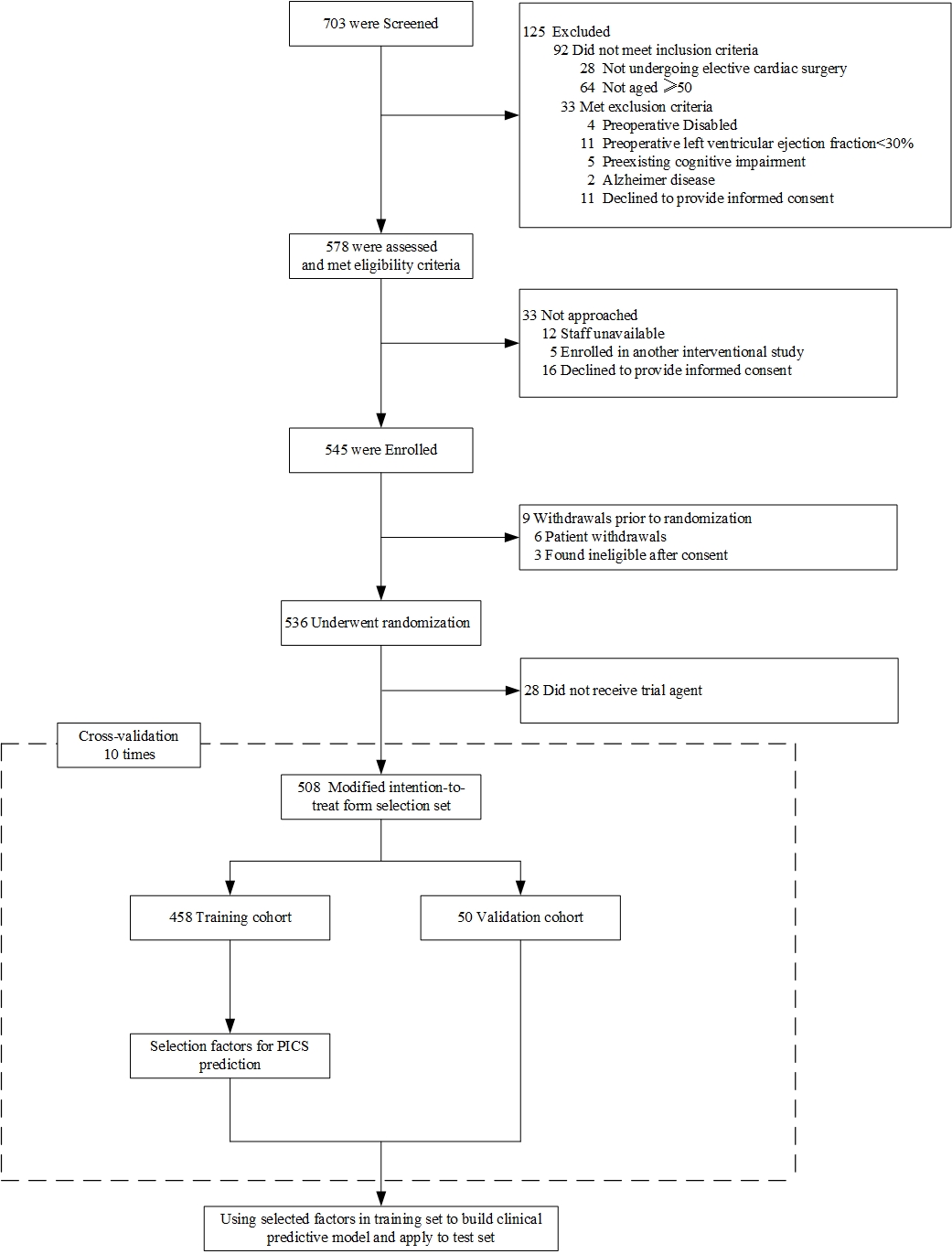


**Figure S4.** **Nomogram model and AUROC for predicting individual risk of psychological impairment in the training cohort. (A)Nomogram model for predicting individual risk of psychological impairment; (B) AUROC for predicting individual risk of psychological impairment in training group; (C) Calibration curves for nomogram-based assessments of psychological impairment in the training cohort***

**(A)**

**
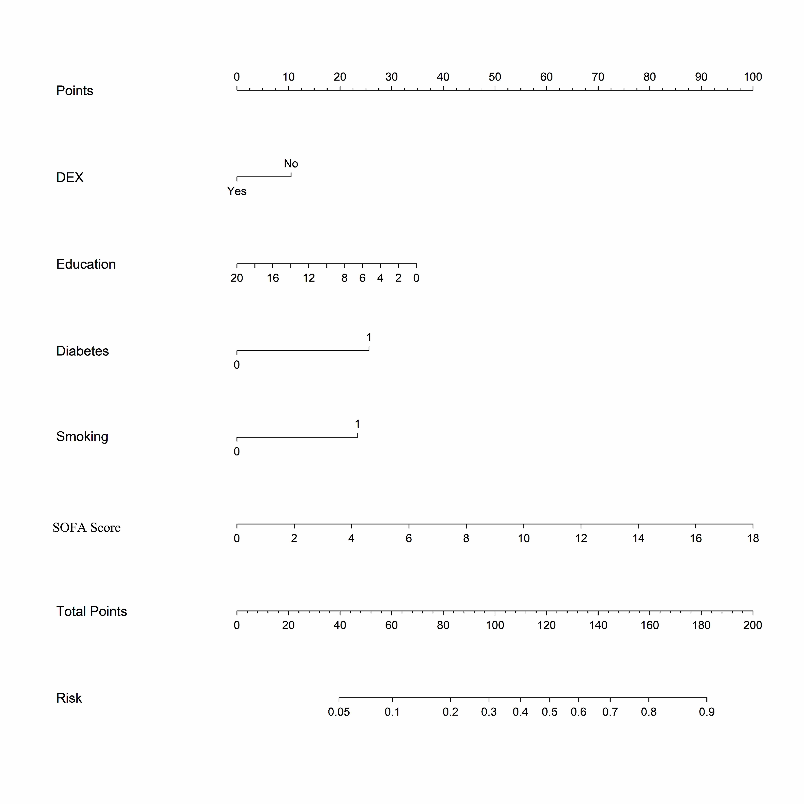
**

**(B)**

**
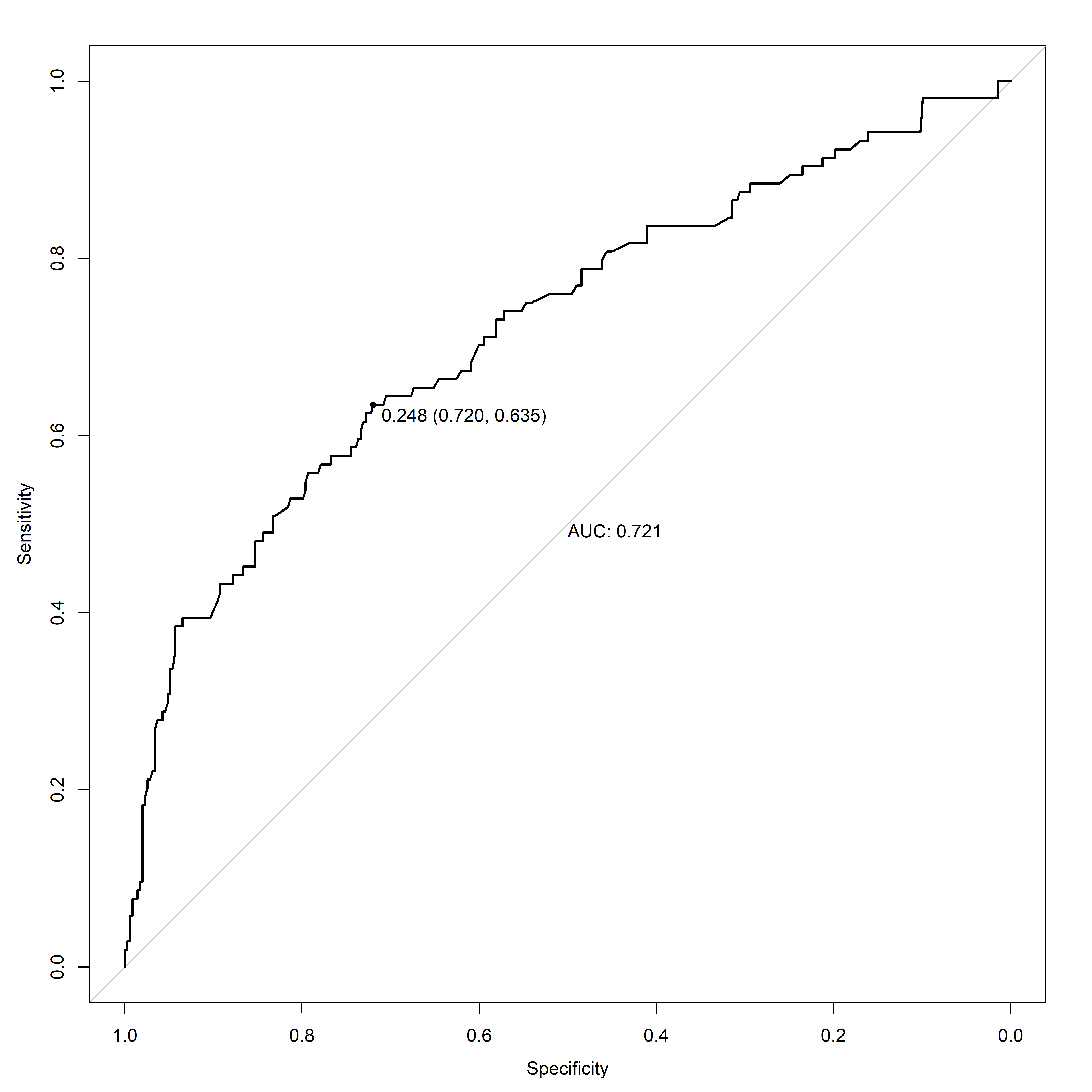
**

**(C)**

**
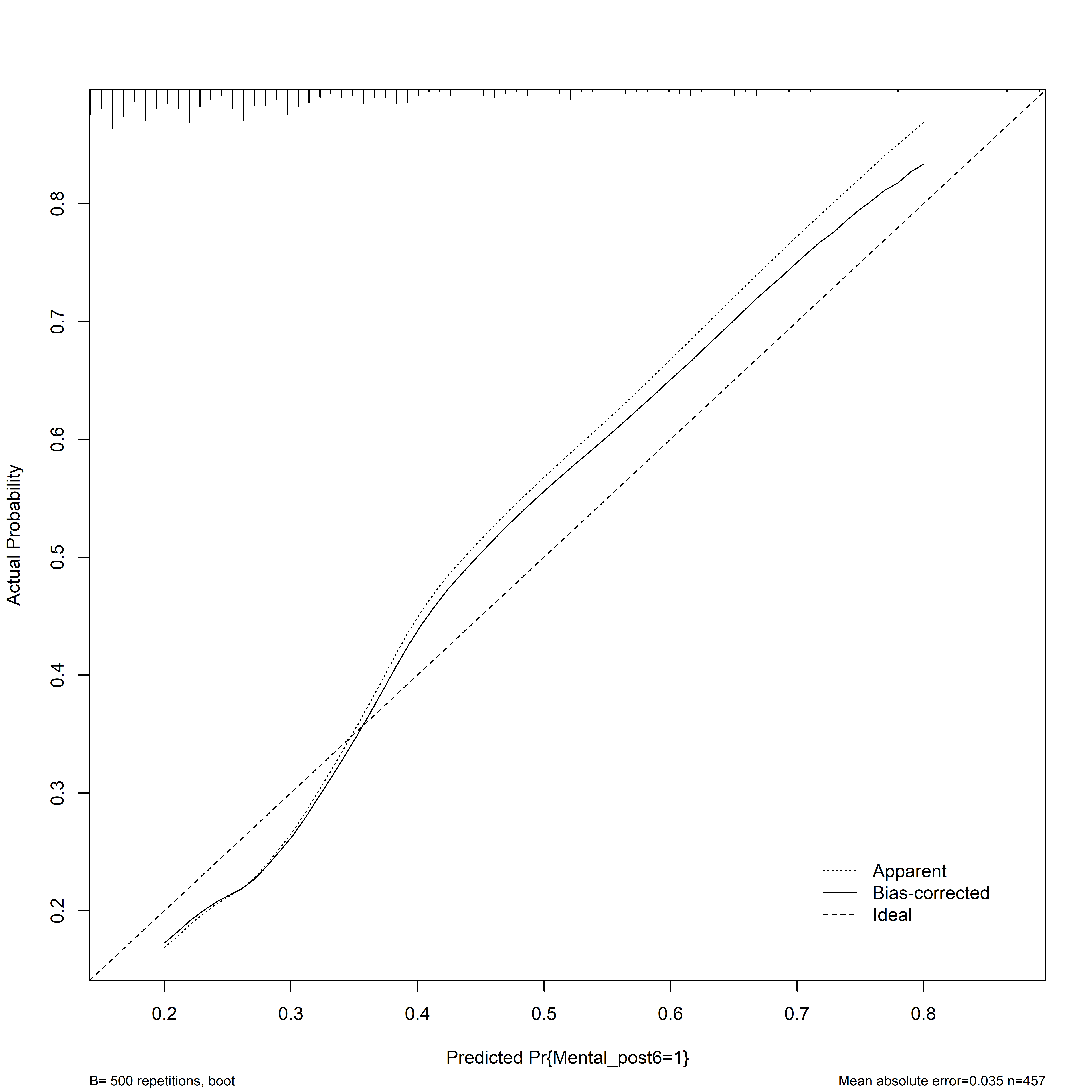
**

* Low incidence of physical and cognitive impairs occurred in 6-moths follow-up resulted in a not good agreement between actual and predicted odds of calibration plots, we did not develop the nomogram of physical and cognitive impairs. *DEX* dexmedetomidine, *SOFA score* The Sequential Organ Failure Assessment score
