# Supplementary figures and images for: Nocturnal dexmedetomidine alleviates post–intensive care syndrome following cardiac surgery: a prospective randomized controlled clinical trial
Source: BMC Med. 2021 Dec 6;19:306. doi: 10.1186/s12916-021-02175-2 (PMC8647374; doi:10.1186/s12916-021-02175-2)

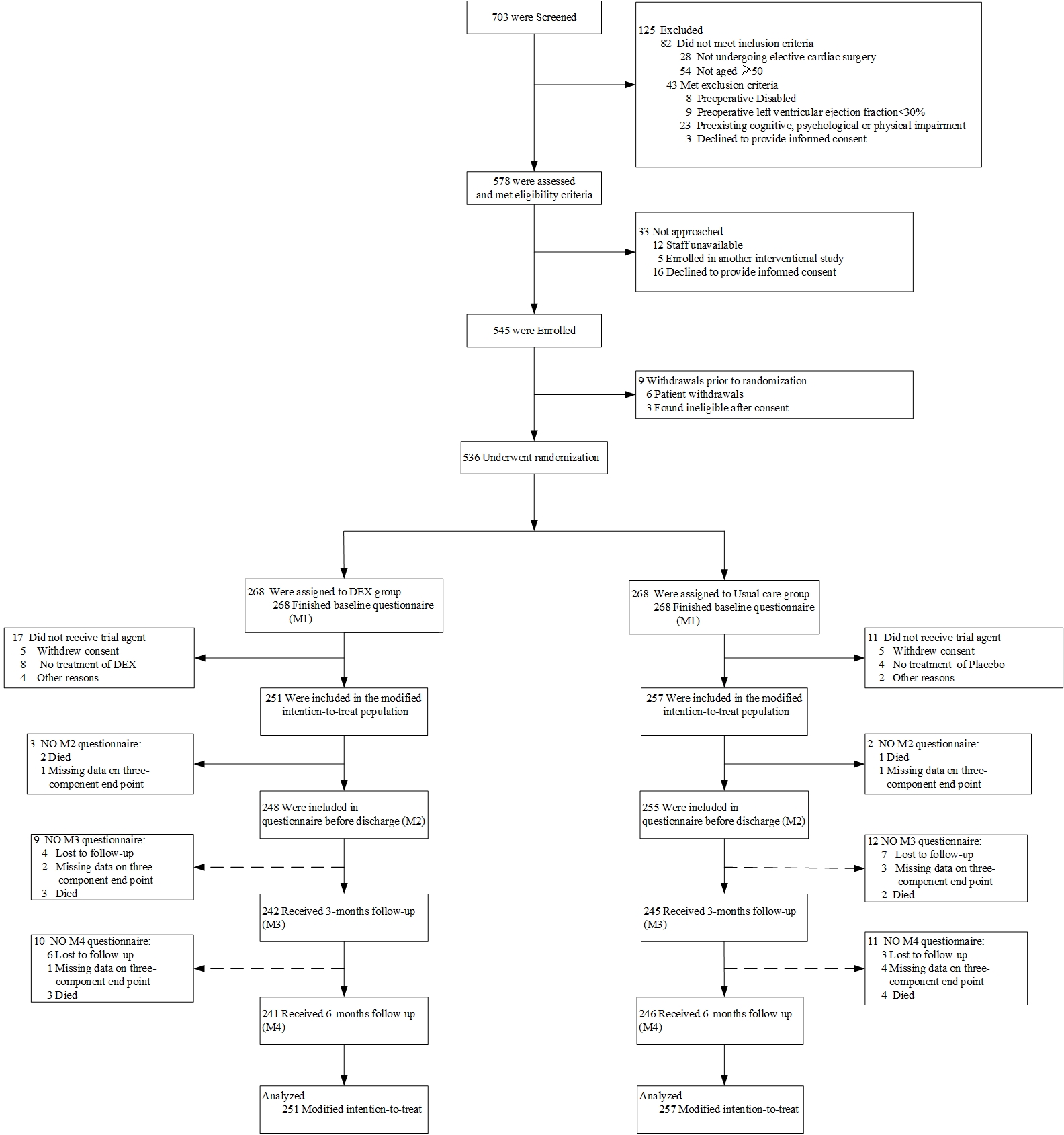

Supplement: Supplementary file 2 — Additional file 2. [file 12916_2021_2175_MOESM2_ESM.jpg]

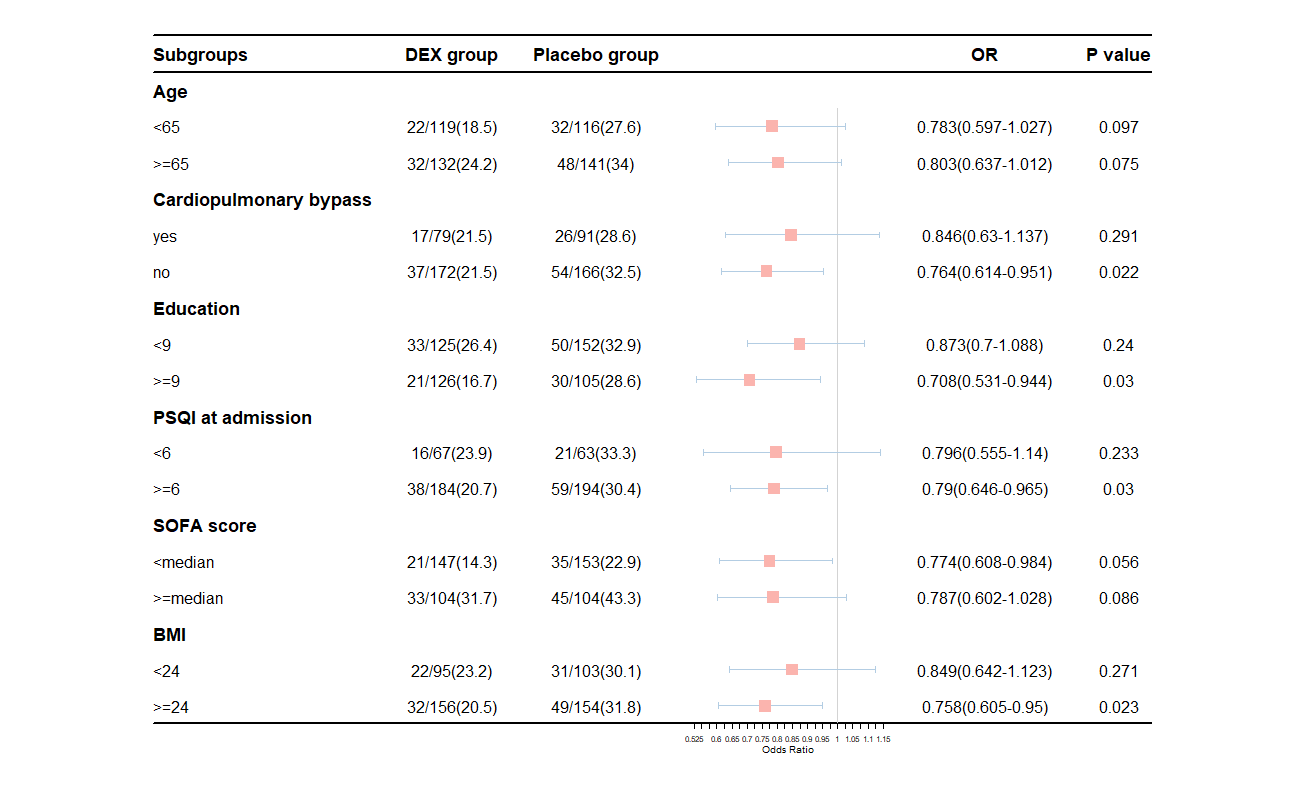

Supplement: Supplementary file 3 — Additional file 3. [file 12916_2021_2175_MOESM3_ESM.tiff]

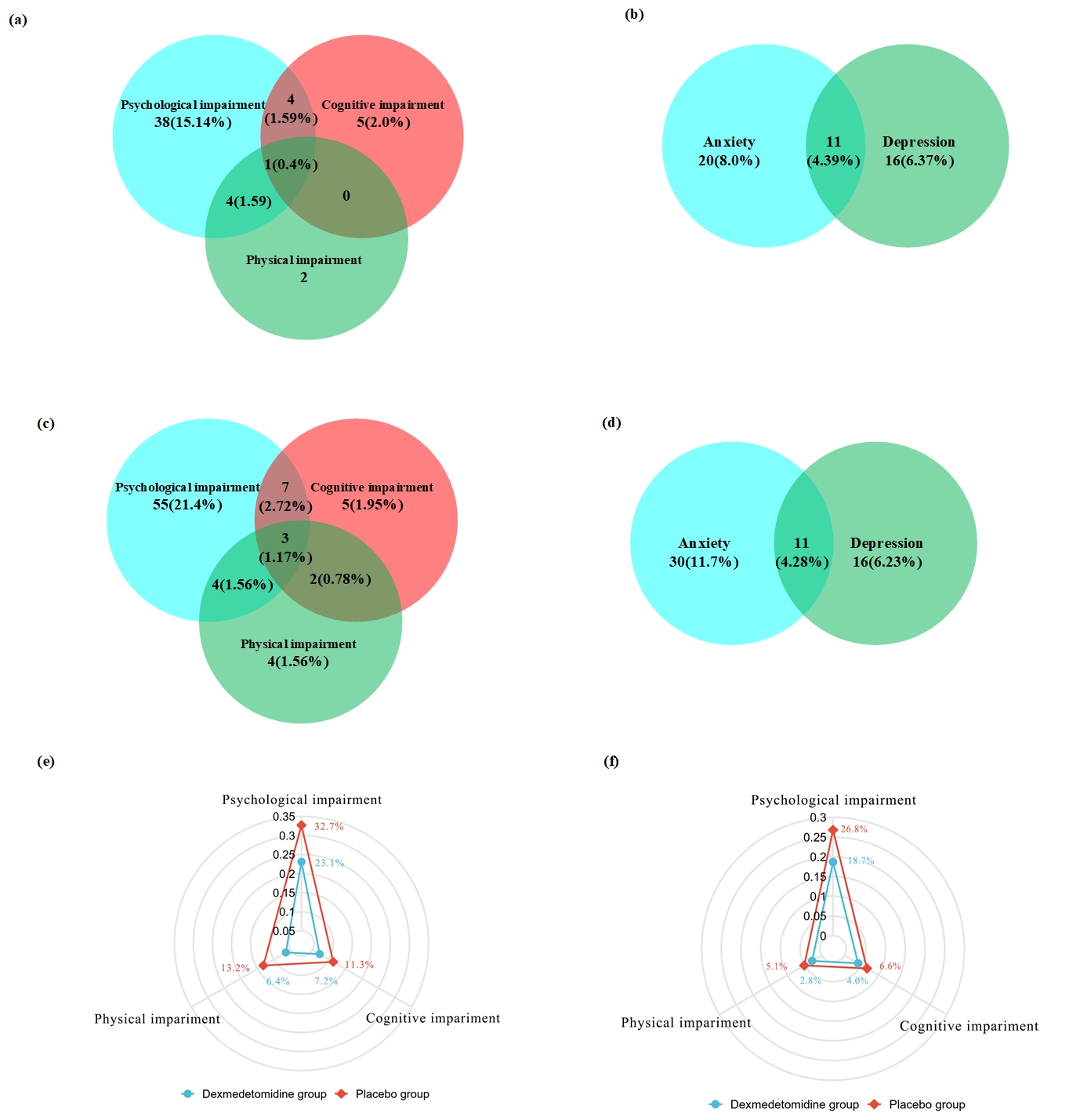

Supplement: Supplementary file 4 — Additional file 4. [file 12916_2021_2175_MOESM4_ESM.jpg]

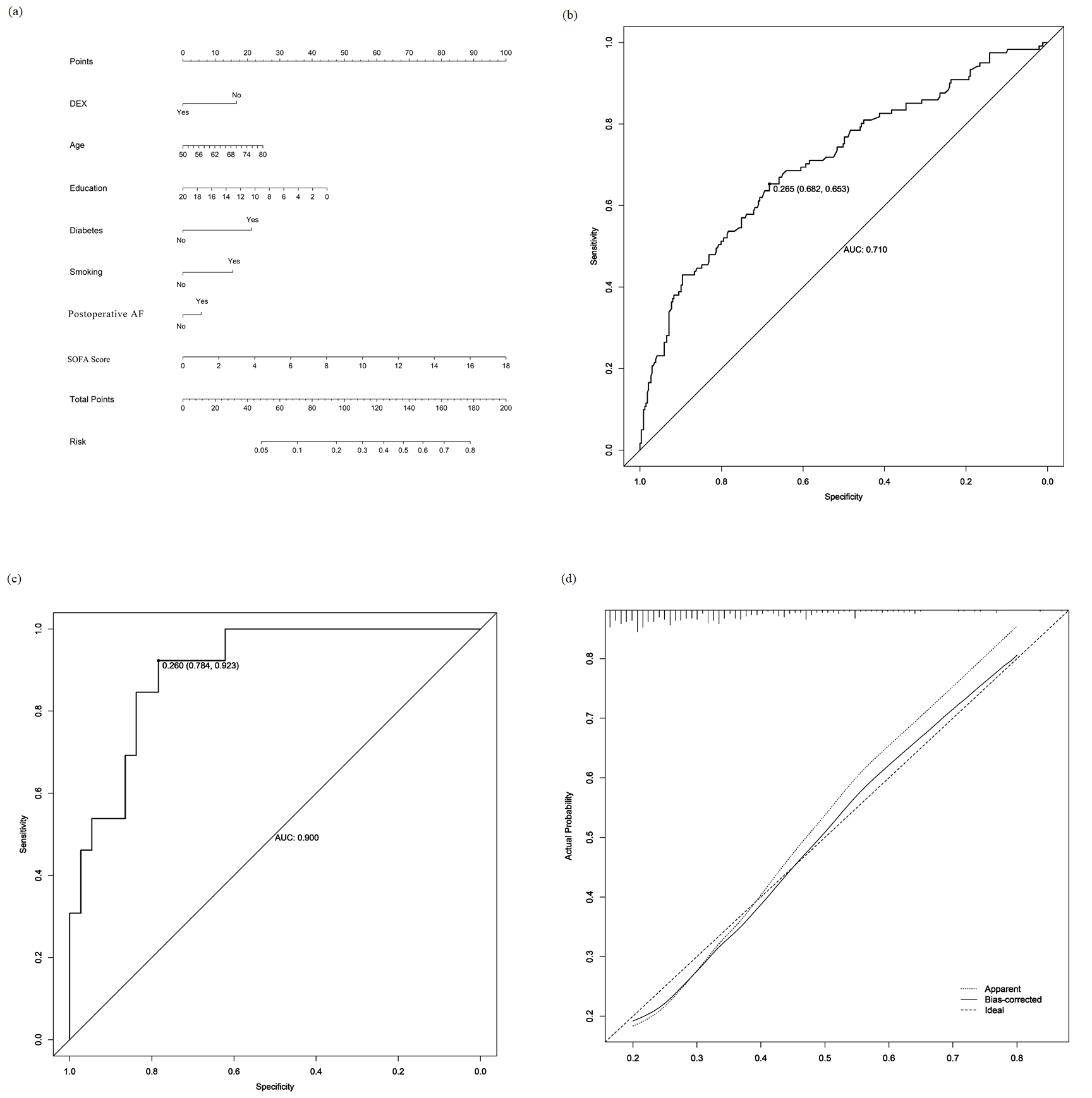

Supplement: Supplementary file 5 — Additional file 5. [file 12916_2021_2175_MOESM5_ESM.jpg]

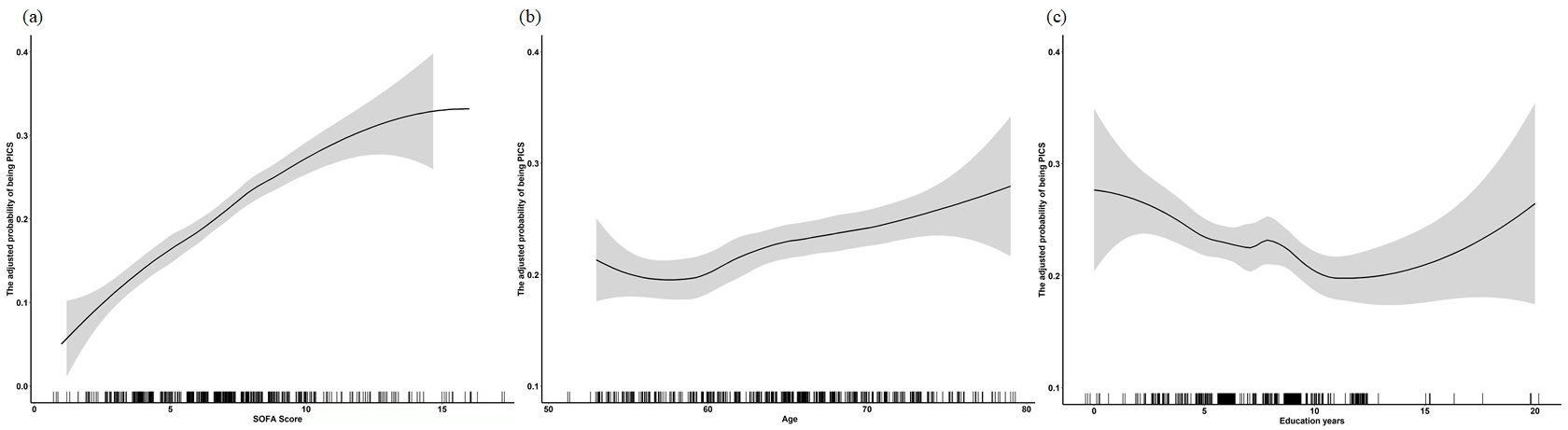

Supplement: Supplementary file 6 — Additional file 6. [file 12916_2021_2175_MOESM6_ESM.jpg]
