# Supplementary material for: Nocturnal dexmedetomidine alleviates post–intensive care syndrome following cardiac surgery: a prospective randomized controlled clinical trial
Source: BMC Med. 2021 Dec 6;19:306. doi: 10.1186/s12916-021-02175-2 (PMC8647374; doi:10.1186/s12916-021-02175-2)
Supplement: Supplementary file 7 — Additional file 7. [file 12916_2021_2175_MOESM7_ESM.docx]

| Table1. Baseline demographics and characteristics | |  |  |
| --- | --- | --- | --- |
| Variables | Dexmedetomidine group(n=251) | Placebo group(n=257) | p |
| Age, mean±SD, yr | 64.84±6.14 | 64.57±6.22 | 0.624 |
| BMI, mean±SD, kg/m^2^ | 24.98±3.00 | 24.87±3.26 | 0.695 |
| Surgery classification, n (%) |  |  | 0.347 |
| without CPB | 172（68.5%） | 166（64.6%） |  |
| With CPB | 79（31.5%） | 84（35.4%） |  |
| Men, n (%) | 166（66.1%） | 156（60.7%） | 0.204 |
| Education, mean±SD, yr | 7.87±2.847 | 7.46±3.276 | 0.133 |
| Hypertension, n (%) | 132(52.6%) | 134(52.1%) | 0.919 |
| Diabetes, n (%) | 76(30.3%) | 77(30.0%) | 0.938 |
| Renal failure, n (%) | 6(2.4%) | 5(1.9%) | 0.731 |
| Infarction, n (%) | 40(15.9%) | 38(14.8%) | 0.719 |
| Smoking, n (%) | 75(29.9%) | 89(34.6%) | 0.252 |
| Alcohol, n (%) | 61(24.3%) | 60(23.3%) | 0.8 |
| LVEF, n (%) |  |  | 0.692 |
| ≤40% | 12（4.8%） | 13（5.1%） |  |
| 41%-60% | 200(79.7%) | 197(76.7%) |  |
| ≥61% | 39(15.5%) | 47(18.3%) |  |
| Atrial fibrillation before surgery | 13(52%) | 11(4.3%) | 0.633 |
| SOFA score at 8 h after surgery ^a^ | 7.16±3.1 | 7.12±3.01 | 0.899 |
| APACHE Ⅱ at 8 h after surgery ^b^ | 7.706±2.15 | 7.63±2.012 | 0.686 |
| PSQI score at admission ^c^ | 7.1±2.121 | 7..06±2.161 | 0.844 |

*BMI* Body mass index, *CPB* Cardiopulmonary bypass, *COPD* chronic obstructive pulmonary disease, *LVEF* Left ventricular ejection fraction, *SOFA* sequential organ failure assessment, *APACHE II* Acute Physiology and Chronic Health Evaluation II, PSQI

^a^ SOFA scores are based on six scores, with respiratory, cardiovascular, hepatic, coagulation, renal and neurological system function being scored from 0-4 where higher scores correspond to more severe organ dysfunction

^b^ APACHE Ⅱ scores (0-71) are based upon the values of 12 routine physiological measurements, age, and prior health status, providing a general measurement of disease severity

^c^ The PSQI questionnaire consists of 19 scored items assessing seven factors: subjective sleep quality, sleep latency, sleep duration, habitual sleep efficiency, sleep disturbances, use of sleeping pills, and daytime dysfunction. All factors are scored from 0-3, with higher scores corresponding to poorer quality of sleep.
