# Supplementary material for: Nocturnal dexmedetomidine alleviates post–intensive care syndrome following cardiac surgery: a prospective randomized controlled clinical trial
Source: BMC Med. 2021 Dec 6;19:306. doi: 10.1186/s12916-021-02175-2 (PMC8647374; doi:10.1186/s12916-021-02175-2)
Supplement: Supplementary file 8 — Additional file 8. [file 12916_2021_2175_MOESM8_ESM.docx]

Table 2. Primary and secondary endpoints

| Variables | | Dexmedetomidine group (n=251) | | Placebo group(n=257) | | OR (95%CI) | P value | |  |
| --- | --- | --- | --- | --- | --- | --- | --- | --- | --- |
| **Primary endpoint** | |  | |  | |  |  | |  |
| PICS at 6-month after discharge ^#^ | | 54（21.5%） | | 80（31.1%） | | 0.793（0.665-0.945） | 0.014 | |  |
| Individual component of the primary end point | | | | |  | | |  | |
| Cognitive impairment by MMSE ^a^ | 10（4.0%） | | 17（6.6%） | | 0.792（0.585-1.073） | | | 0.186 | |
| Psychological impairment by SAS and SDS ^b,c#^ | 47（18.7%） | | 69（26.8%） | | 0.806（0.672-0.967） | | | 0.029 | |
| Physical impairment by Barthel index ^d^ | 7（2.8%） | | 13（5.1%） | | 0.769（0.551-1.074） | | | 0.188 | |
| **Secondary endpoints** | |  | |  | |  |  | |  |
| Mortality in hospitalization, No (%) | | 2（0.8%） | | 1（0.4%） | | 1.521（0.306-7.522） | 0.549 | |  |
| Mortality within 6 months, No (%) | | 3（1.2%） | | 4（1.6%） | | 0.884（0.463-1.688） | 0.727 | |  |
| PICS at 3-month after discharge ^#^ | | 79 (31.5%) | | 110 (42.8%) | | 0.792(0.668-0.938) | 0.008 | |  |
| Cognitive impairment* | | 18(7.2%) | | 29(11.3%) | | 0.802(0.628-1.022) | 0.11 | |  |
| Psychological impairment^*,#^ | | 58(23.1%) | | 84(32.7%) | | 0.799(0.671-0.951) | 0.016 | |  |
| Disability^*,#^ | | 16(6.4%) | | 34(13.2%) | | 0.716(0.579-0.885) | 0.01 | |  |
| ICU stay | | 3.0（2.0,4.0） | | 3.0（2.0,4.0） | | - | 0.642 | |  |
| Length of hospital stay | | 8（7.0,10.0） | | 8（7.0,11.0） | | - | 0.157 | |  |
| Tracheal intubation time | | 13（9,16） | | 13（9,17） | | - | 0.346 | |  |
| Retracheal intubation | | 2（0.8%） | | 5（1.9%） | | 0.704（0.437-1.134） | 0.267 | |  |
| Acute kidney injury | | 3（1.2%） | | 2（0.8%） | | 1.267（0.432-3.721） | 0.634 | |  |
| delirium | | 19 (7.6%) | | 31 (12.1%) | | 0.796(0.629-1.008) | 0.089 | |  |
| Postoperative atrial fibrillation | | 57 (22.7%) | | 80 (31.1%) | | 0.817(0.684-0.975) | 0.033 | |  |
| Safety Outcomes | |  | |  | |  |  | |  |
| Any adverse event occurred ^#^ | | 45（17.9%） | | 26（10.1%） | | 1.443(1.050-1.985) | 0.011 | |  |
| Hypotension after treatment initiation ^#^ | | 32（12.7%） | | 17（6.6%） | | 1.507(1.016-2.235) | 0.019 | |  |
| Bradycardia after treatment | | 17（6.8%） | | 12（4.7%） | | 1.236(0.795-1.923) | 0.307 | |  |
| Extra fluid intervention | | 13（5.2%） | | 6（2.3%） | | 1.625(0.834-3.168) | 0.091 | |  |
| Extra vasoconstrictor intervention | | 7（2.8%） | | 4（1.6%） | | 1.4(0.638-3.074) | 0.34 | |  |

Data are given as means ± standard deviation (SD) or mean (IQR) for measurement variables, and number of patients (n) and percentages (%) for categorical variables. *OR* odds ratio, *CI* confidence interval, *PICS* post–intensive care syndrome, *MMSE* mini-mental state examination, *SAS* Zung’s Self-Rating Anxiety Scale, *SDS* Self-Rating Depression Scale

^a^ The Mini-Mental State Examination (MMSE) uses a 30-point scale to evaluate cognitive function based upon tests of patient orientation, concentration, attention, verbal memory, naming and visuospatial skills. Scores of < 27 points are consistent with potential cognitive impairment.

^b^ Zung’s SAS is a 20-item questionnaire with scores ranging from 20-80 points. A score of > 50 points is consistent with potential generalized anxiety disorder.

^c^ Zung’s SDS contains 20 items using a 4-point Likert scale. The raw sum score of the SDS ranges from 20 to 80 but results are usually presented as the SDS Index, which is obtained by expressing the raw score is converted to 100 points scale. The SDS cut-off points for depression was > 50 based on literature.

^d^ The Barthel Index is a scale that assesses the ability to perform particular activities of daily living. This index consists of 10 tasks that are scored from 0-100, with higher scores corresponding to greater mobility. A score of < 80 is consistent with potential physical impairment.

* rates of cognitive impairment, anxiety and disability was at 3 months follow-up

# variables with significant difference, p ˂0.05
